# Effects of Mental Health Interventions on Depressive Symptoms, Anxiety, and Sleep Quality in University Students: A Systematic Review and Meta-Analysis

**DOI:** 10.3390/jcm15197630

**Published:** 2026-10-01

**Authors:** Josefa Salazar-Diaz, Edgar Vásquez-Carrasco, Jordan Hernandez-Martinez, Pablo Aravena-Sagardía, Roberto Lopez-Andaur, Sebastián Burgos-Carrasco, Eduardo José Fernández-Rodríguez, Cristian Sandoval-Vásquez, Pablo Valdés-Badilla

**Affiliations:** 1 School of Occupational Therapy, Faculty of Psychology, Universidad de Talca, Talca 3465548, Chile; jsalazar22@alumnos.utalca.cl (J.S.-D.); edgar.vasquez@utalca.cl (E.V.-C.); 2 Centro de Investigación en Ciencias Cognitivas, Facultad de Psicología, Universidad de Talca, Talca 3465548, Chile; 3 VITALIS Longevity Center, Universidad de Talca, Talca 3465548, Chile; 4 Department of Physical Activity Sciences, Universidad de Los Lagos, Osorno 5290000, Chile; jordan.hernandez@ulagos.cl; 5 Department of Education, Faculty of Humanities, Universidad de La Serena, La Serena 1700000, Chile; 6 Physical Education Pedagogy, Faculty of Education, Universidad Autónoma de Chile, Temuco 4810101, Chile; pablo.aravena@uautonoma.cl; 7 Exercise and Rehabilitation Sciences Institute, School of Occupational Therapy, Faculty of Rehabilitation Sciences, Universidad Andres Bello, Santiago de Chile 7591538, Chile; r.lopezandaur@uandresbello.edu; 8 Académico Escuela de Terapia Ocupacional, Facultad de Salud y Ciencias Sociales, Universidad de Las Américas, Concepción 4030000, Chile; sburgos@udla.cl; 9 Institute of Biomedical Research of Salamanca (IBSAL), 37007 Salamanca, Spain; 10 Department of Nursing and Physiotherapy, University of Salamanca, 37007 Salamanca, Spain; 11 Escuela de Tecnología Médica, Facultad de Salud, Universidad Santo Tomás, Los Carreras 753, Osorno 5310431, Chile; 12 Departamento de Medicina Interna, Facultad de Medicina, Universidad de La Frontera, Temuco 4811230, Chile; 13 Department of Physical Activity Sciences, Faculty of Education Sciences, Universidad Católica del Maule, Talca 3530000, Chile; 14 Sports Coach Career, School of Education, Universidad Viña del Mar, Viña del Mar 2520000, Chile

**Keywords:** depression, insomnia, non-pharmacological interventions, university student, undergraduate student

## Abstract

**Background**: Mental health problems are common among university students. This systematic review and meta-analysis examined the effects of mental health interventions on depressive symptoms, anxiety, and sleep quality in this population. **Methods**: Randomized controlled trials published through 30 July 2026 were identified in MEDLINE/PubMed, Scopus, the Cochrane Library, Web of Science Core Collection, EBSCOhost, CINAHL, and ProQuest. Risk of bias and certainty of evidence were assessed using Cochrane Risk of Bias 2 and GRADE, respectively. Random-effects meta-analyses used mean differences (MDs) for outcomes reported on the same scale and Hedges’ g for sleep outcomes that used non-equivalent PSQI scores. Between-study variance was estimated using restricted maximum likelihood, with modified Hartung Knapp Sidik Jonkman inference. The protocol was registered in PROSPERO (CRD420261344616). **Results**: Nine studies met the eligibility criteria, and seven contributed to at least one meta-analysis. Interventions reduced PHQ-9 scores on average (MD = 2.21 points, 95% CI 0.89 to 3.54; *p* = 0.006; I^2^ = 81.0%), although the 95% prediction interval included no effect (−1.20 to 5.63). The pooled effects on GAD-7 scores (MD = 1.99, 95% CI −0.48 to 4.46; *p* = 0.089; I^2^ = 92.0%) and sleep quality (Hedges’ g = 1.30, 95% CI −3.08 to 5.68; *p* = 0.330; I^2^ = 99.1%) were not statistically conclusive. All prediction intervals included the null. **Conclusions**: Mental health interventions may reduce depressive symptoms among university students on average, but substantial heterogeneity limits the generalizability of this finding. Evidence for effects on anxiety and sleep quality remains inconclusive. More rigorous and clinically comparable trials are required.

## 1. Introduction

University years are associated with high levels of depression symptoms, anxiety, and poor sleep [1,2]. For many students, it is also a time of transition in terms of academic workload, social relationships and day-to-day routines. These demands can contribute to the mental health burden [3,4]. Depression is particularly relevant as symptoms can impact motivation, daily functioning and academic performance [5]. The student groups’ symptoms are strongly related to sleep [6,7]. The relationship is likely to be bidirectional, with poor sleep potentially preceding or worsening depressive symptoms, and depression potentially disrupting sleep continuity and regulation [8,9]. This is a very common combination and there are functional consequences. The availability of early support is an important consideration in higher education [10].

Anxiety is another aspect of this problem, which can co-occur with depressive symptoms [1,11]. The pressures of university are multifaceted. Workload and examinations are obvious sources of stress but worry and arousal can be maintained by uncertainty about performance, social adjustment and limited support [4]. For some students these problems continue into the night and impact their ability to fall or stay asleep [8,9]. So, sleep problems overlap with depression and anxiety, but are not the same as depression and anxiety. Therefore, it is best to look at their response to intervention separately.

Sleep itself is more than a secondary concern. Sleep problems are a common problem among university students [12,13]. The consequences are not limited to feeling tired. Attention, memory, emotional control and recovery may be impaired, having consequences in the daily life and academic performance of the students [6,14,15]. Getting regular sleep can be difficult when you are at university. Assessments can add to overall workload, digital engagement can stretch to late hours, and schedules are often irregular [13,16]. Some students report symptoms of insomnia, i.e., difficulty falling asleep, staying asleep or feeling rested after sleep [17]. Persistent difficulties in emotional regulation, maintaining cognitive or physiological arousal, can lead to depression and anxiety [8,14].

Many ways have been considered to solve these many difficulties. The college students who engaged in psychological therapies had fewer depression symptoms and better mental health outcomes [2,18,19,20]. Digital delivery is increasingly important. For example, internet-delivered cognitive behavioral therapy has been shown to be effective for anxiety and may offer help outside of traditional face-to-face treatment [21]. Other methods are more directly related to sleep and have demonstrated increases in sleep quality, sometimes with psychological benefits [22,23]. Some evidence from people with depression suggests that non-pharmacological therapies may improve sleep and depression symptoms. Cognitive behavioral techniques, in particular, may be helpful [24].

Previous reviews of university mental health have generally synthesized broad and heterogeneous outcome sets [2,19,20] or focused specifically on anxiety [21] or sleep [22,23]. Consequently, they do not provide an RCT-only synthesis of depressive symptoms, anxiety, and sleep quality using outcome-specific instruments and a common framework for RoB 2 and GRADE. The present review addresses this gap by evaluating immediate post-intervention effects on depressive symptoms as the primary outcome and anxiety and sleep quality when co-reported, while examining robustness, heterogeneity, adherence, safety, risk of bias, and certainty of evidence. Each outcome was synthesized separately; their parallel evaluation was intended to compare the strength and consistency of evidence across domains and does not imply a common mechanism or justify pooling clinically distinct interventions.

## 2. Materials and Methods

### 2.1. Protocol and Registration

This review followed the Cochrane methodological guidance and was reported according to the PRISMA 2020 statement [25] (Supplementary Table S1). The protocol was registered in PROSPERO on 18 March 2026, after formal searching had begun but before study screening, data extraction, risk-of-bias assessment, or evidence synthesis (CRD420261344616; https://www.crd.york.ac.uk/PROSPERO/view/CRD420261344616; accessed on 30 September 2026). The registration specified RCTs of mental health treatment versus usual care in university students aged ≥18 years with diagnosed depression, with depressive symptoms and sleep quality as main outcomes; no additional outcomes, assessment time points, or detailed quantitative synthesis methods were prespecified. During the review, eligibility was broadened to encompass the prevention-to-treatment continuum and active or inactive comparators. Depressive symptoms were required for inclusion, whereas anxiety and sleep quality were treated as secondary outcomes. Information sources were expanded to improve search sensitivity, and the final search was completed on 30 July 2026.

### 2.2. Eligibility Criteria

Peer-reviewed RCTs available through the final search date were eligible without restrictions on publication year or language. Using PICOS, we included university or college students aged ≥18 years, structured non-pharmacological interventions with an explicit preventive or therapeutic mental health or sleep-related aim, any randomized active or inactive comparator, and depressive symptoms assessed with a validated instrument at baseline and at least once after randomization. Elevated symptoms or a formal diagnosis of depression were not required. Anxiety and sleep quality were secondary outcomes when co-reported and were insufficient alone for eligibility.

For descriptive synthesis, samples were classified as universal, selective, indicated, or treatment populations, while interventions were categorized by predominant content as cognitive–behavioral/problem-solving, mindfulness/acceptance-based, sleep-focused/psychoeducational, or multicomponent digital. Guidance was classified as guided or self-guided. These classifications characterized clinical heterogeneity, did not determine eligibility, and did not imply equivalence among interventions. Full eligibility criteria are presented in Table 1.

### 2.3. Information Sources and Search Strategy

A comprehensive literature search was conducted in MEDLINE (via PubMed), Scopus (Elsevier), the Cochrane Central Register of Controlled Trials (CENTRAL; via the Cochrane Library), Web of Science Core Collection (Clarivate), APA PsycINFO and CINAHL Complete (via EBSCOhost), and ProQuest Central (ProQuest). The databases were initially searched in November 2025 and last updated on 30 July 2026, without publication-date or language filters. Controlled vocabulary and free-text terms were used to identify structured mental health interventions reporting depression, anxiety, or sleep-related outcomes in university students. The broad outcome block maximized search sensitivity; however, eligibility required depressive symptoms to be assessed using a validated instrument after randomization, whereas anxiety and sleep outcomes alone were insufficient for inclusion.

The core search strategy was: (“university student” OR “college student” OR “undergraduate student” OR “graduate student” OR “postgraduate student” OR “higher education student” OR “tertiary education student”) AND (“depression” OR “depressive disorder” OR “major depressive disorder” OR “depressive symptoms” OR “major depression” OR “anxiety” OR “anxiety disorder” OR “anxiety symptoms” OR “generalized anxiety disorder” OR “psychological distress” OR “sleep quality” OR insomnia OR “sleep disturbance” OR “sleep problem” OR “sleep disorder”) AND (“mental health intervention” OR intervention OR treatment OR psychotherapy OR “cognitive behavioral therapy” OR mindfulness OR “digital intervention” OR “internet-based intervention” OR “mobile intervention”). This strategy was adapted to each database using the appropriate field tags and a randomized-trial block where applicable. The complete strategies, platforms, limits, exact search date, and source-specific yields are provided in Supplementary Table S2. Reference lists, forward citations, trial registries, dissertations, preprints, and unpublished or ongoing studies were not searched.

### 2.4. Study Selection

All retrieved records were imported into Mendeley Reference Manager (version 2.116.3; Elsevier, Amsterdam, The Netherlands). After duplicate removal, two reviewers independently screened titles and abstracts and subsequently assessed potentially eligible full-text articles against the predefined eligibility criteria. Reasons for full-text exclusion were documented. A third reviewer independently audited the study selection process. The overall selection process was summarized using a PRISMA flow diagram.

### 2.5. Data Extraction

Data were extracted using a predefined Microsoft Excel form (version 16.81; Microsoft Corporation, Redmond, WA, USA), developed in accordance with guidance from the Cochrane Handbook [26]. Two investigators independently extracted data, and a third investigator verified the information for consistency and accuracy. Extracted data included study and methodological characteristics, participant characteristics, intervention and comparator conditions, intervention-related variables, outcome measures, and reported findings.

### 2.6. Risk of Bias

Risk of bias was assessed using the Cochrane Risk of Bias 2 (RoB 2) tool [26], targeting the effect of assignment to intervention irrespective of adherence. Five domains were evaluated: bias arising from the randomization process; bias due to deviations from intended interventions; bias due to missing outcome data; bias in measurement of the outcome; and bias in selection of the reported result. The RoB 2 signaling questions and algorithms were followed to derive domain-level and overall judgments of low risk, some concerns, or high risk. Two reviewers independently completed the assessments; disagreements were resolved through consensus, and a third reviewer verified the final judgments.

### 2.7. Meta-Analysis

Detailed quantitative synthesis procedures were not specified in the PROSPERO registration; therefore, the analytic decisions described below were post-registration operational decisions. Analyses were performed in Python 3.12.14 using NumPy 2.3.5 and SciPy 1.17.0, with figures generated using Matplotlib 3.10.8. The extraction dataset and executable code were retained to support reproducibility.

For each outcome, one estimate per independent comparison was selected at the end of treatment or, when first measured later, at the earliest available post intervention assessment. Intention-to-treat and trial-reported adjusted estimates were preferred; otherwise, available-case data and postintervention group means, standard deviations, and sample sizes were used. Standard errors were derived from reported confidence intervals when necessary. No standard deviations were imputed, and author contact was unnecessary because the required data were available or derivable. Eligible intervention arms in multi-arm trials were combined using Cochrane formulas to avoid counting a shared control group more than once. Effects on PHQ-9 and GAD-7 were expressed as mean differences, calculated as control minus intervention so that positive values favored intervention. One eligible study remained in the review but was excluded from the PHQ-9 synthesis because it used the PHQ-8 and no validated conversion was available. Sleep outcomes were expressed as Hedges’ g because two studies reported global PSQI scores and one reported a single PSQI component. Its direction was defined as g = J[(M_C − M_I)/S_p], where J is the small-sample correction. Meta-analysis required at least three clinically compatible studies.

Clinical heterogeneity was examined according to intervention class, human guidance, comparator, recruitment and symptom profile, duration, analysis population, and outcome timing. Random-effects estimates were interpreted as average effects across diverse intervention models, not as evidence that the interventions were interchangeable. Between-study variance was estimated using restricted maximum likelihood, and confidence intervals and *p* values used a modified Hartung–Knapp adjustment with k − 1 degrees of freedom and a safeguard against standard errors smaller than conventional random-effects estimates. Heterogeneity was reported using τ^2^, Cochran’s Q, I^2^, and 95% prediction intervals. Meta-regression and subgroup comparisons were not performed because the available strata were too small. Leave-one-out analyses assessed study influence, whereas funnel-plot tests were omitted because each synthesis contained fewer than 10 studies. The post hoc GOSH analysis was removed because it was not prespecified and was minimally informative with the available number of studies.

### 2.8. Certainty of Evidence

The certainty of the evidence was evaluated separately for each meta-analyzed outcome using the Grading of Recommendations Assessment, Development and Evaluation (GRADE) framework and classified as high, moderate, low, or very low [27]. Because each body of evidence originated from randomized controlled trials, certainty initially started at high and was downgraded by one or two levels, as appropriate, for risk of bias, inconsistency, indirectness, imprecision, or publication bias. Risk-of-bias judgments were based on the outcome-specific RoB 2 assessments of the contributing trials. Inconsistency was evaluated by considering the direction and magnitude of study effects together with τ^2^, I^2^, and the 95% prediction interval. Imprecision was assessed according to the number of contributing studies and participants, the width of the Hartung–Knapp confidence interval, and whether the confidence and prediction intervals included no effect or materially different effects. Publication bias was not assumed to be absent but was assessed qualitatively because fewer than 10 studies contributed to each outcome, precluding reliable funnel-plot asymmetry tests. Two reviewers independently performed the assessments, with disagreements resolved through discussion and consensus. Outcome-specific judgments and reasons for downgrading are reported in the Summary of Findings table.

## 3. Results

### 3.1. Study Selection

**Figure 1:**
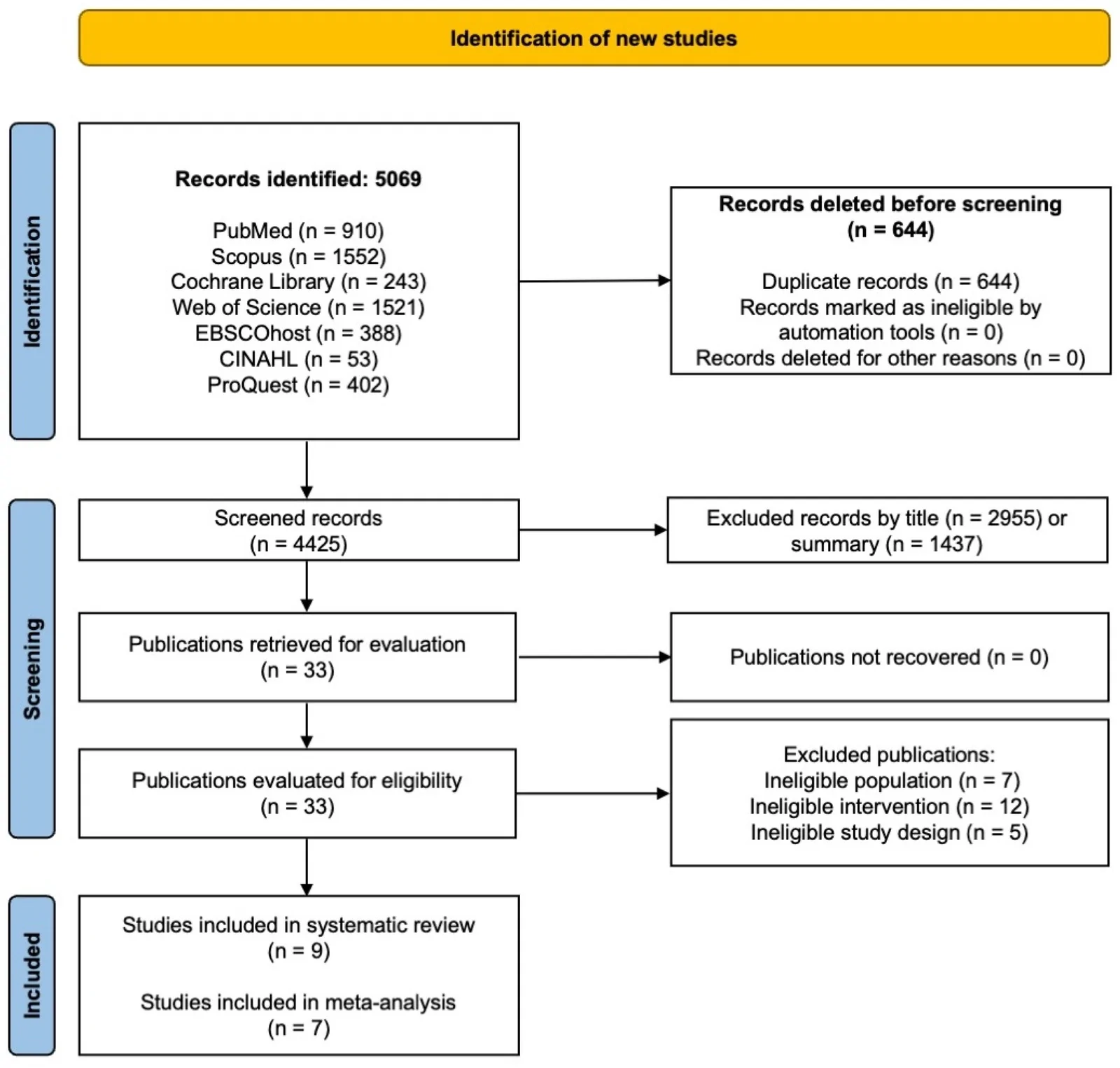
PRISMA 2020 flow diagram illustrating the study selection process [25].

The literature search identified 5069 records, of which 644 were duplicates and removed. The remaining 4425 records were screened by title and abstract, leading to the exclusion of 4392 records (2955 at title screening and 1437 at abstract screening). Thirty-three articles were assessed in full text. Of these, 24 were excluded because of an ineligible population (n = 7), intervention (n = 12), or study design (n = 5). Nine studies therefore met the eligibility criteria and were included in the systematic review [28,29,30,31,32,33,34,35,36]. All nine included trials reported depressive symptoms as an outcome. Anxiety symptoms and sleep quality were available only in subsets of these trials and were therefore treated as secondary outcome domains. Seven provided sufficient data for at least one meta-analysis [29,30,31,32,33,34,35]. The study-selection process is shown in Figure 1.

### 3.2. Risk of Bias

**Figure 2:**
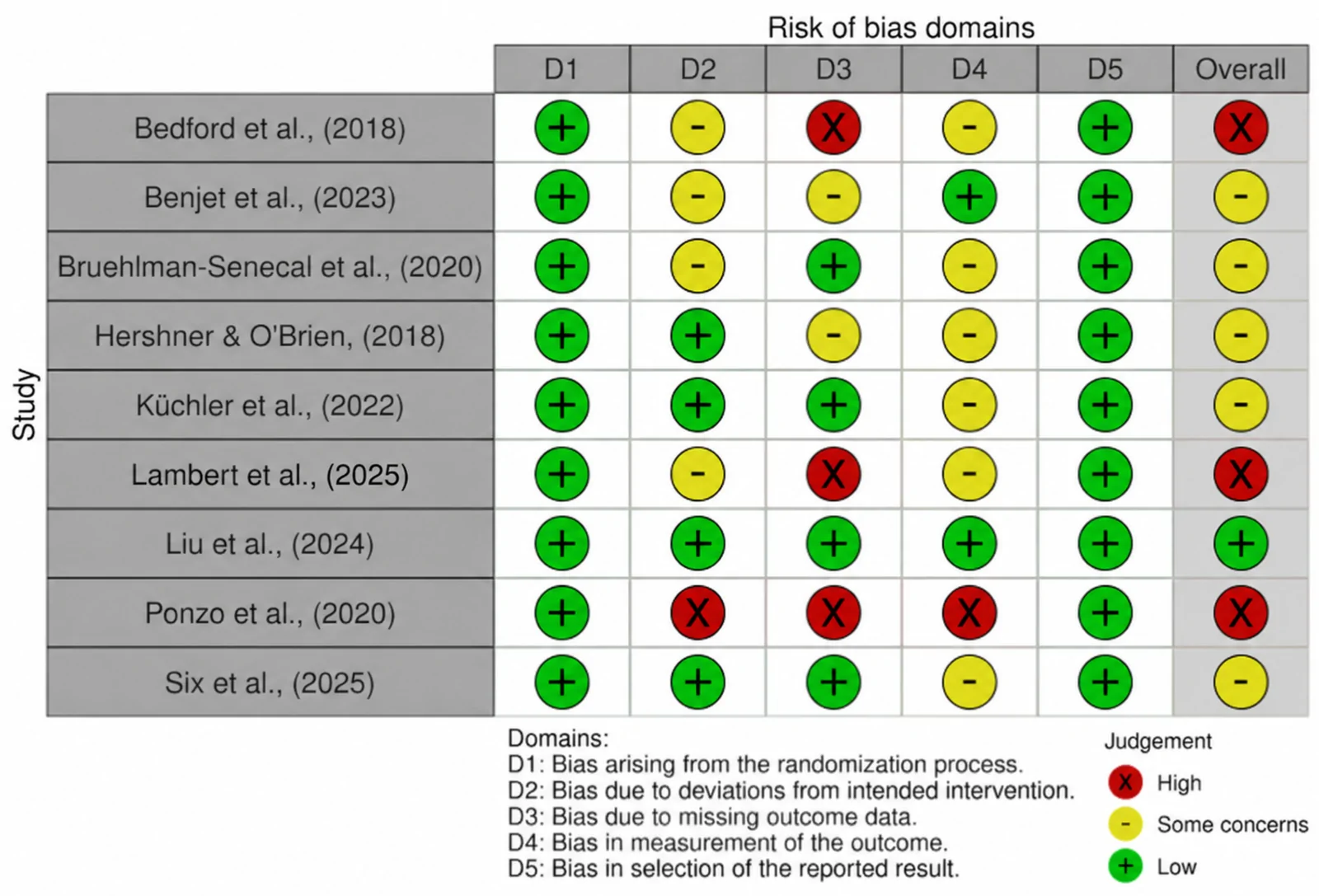
Summary of the risk-of-bias assessments across included studies [28,29,30,31,32,33,34,35,36].

**Figure 3:**
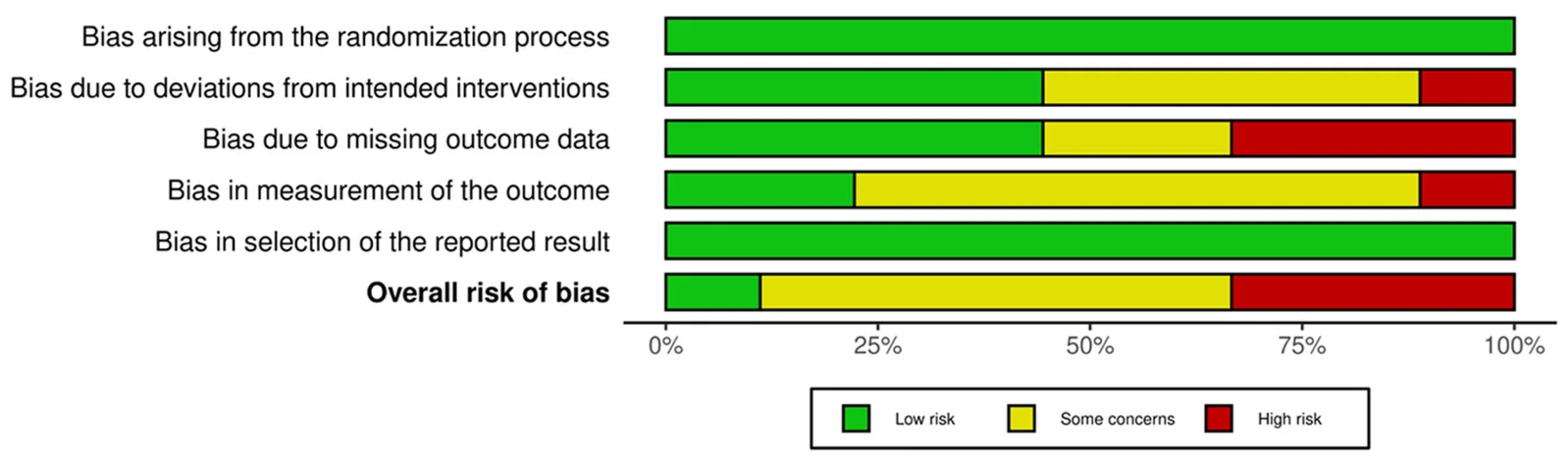
Traffic-light plot of risk-of-bias judgments.

Risk-of-bias judgments are presented in Figure 2 and Figure 3. One study was judged to be at low overall risk of bias [34], five raised some concerns [29,30,31,32,36], and three were judged to be at high risk [28,33,35]. All studies were rated as low risk for the randomization process and selection of the reported result. The high-risk judgments for Bedford et al. [28] and Lambert et al. [33] were driven by missing outcome data, whereas Ponzo et al. [35] was judged to be at high risk because of deviations from intended interventions, missing outcome data, and outcome measurement. Concerns related to the remaining studies were concentrated in deviations from intended interventions, missing outcome data, and measurement of self-reported outcomes. Detailed domain-level risk-of-bias judgments are presented in Figure 2 and Figure 3 and Supplementary Table S3, with supporting rationales for each domain-level judgment provided in Supplementary Table S4.

### 3.3. Characteristics of Included Studies

The nine included RCTs evaluated psychological, behavioral, sleep-focused, and digitally delivered interventions across universal, selective, indicated, and treatment-oriented university-student samples; only Liu et al. [34] required formally diagnosed major depressive disorder. Trials were conducted in the United States, the United Kingdom, China, Colombia, Mexico, Germany, Switzerland, and Austria. Depressive symptoms were assessed with the PHQ-9 in eight studies [28,29,30,31,32,33,34,35] and the PHQ-8 in the study of Six et al. [36]. Five studies used the GAD-7 [29,30,32,33,34], whereas Ponzo et al. [35] assessed anxiety using the STAI-S-6 and DASS-21 anxiety subscale. Sleep was assessed with the global PSQI in two studies [31,34], a PSQI subjective sleep-quality item in the study of Bruehlman-Senecal et al. [30], and the ISI in the study by Bedford et al. [28].

Intervention exposure ranged from a single session [31,33] to eight weeks [29,34]. Other programs comprised six weekly ePST sessions [28], five weekly StudiCare modules with daily practice [32], four weeks of self-directed app access [30,35], and two weeks of alternate-day app use [36]. When reported, sessions lasted approximately 20 min [31], 60–75 min [33], 60 min per module [32], or 150 min [34]. Study samples ranged from 24 to 1319 participants. Across the eight trials reporting a mean age, study-level means ranged from 18.7 to 32.7 years; Liu et al. [34] reported age categories of 19–29 years rather than a mean.

Four trials primarily evaluated cognitive–behavioral or problem-solving content [28,29,33,36], two evaluated mindfulness- or acceptance-based programs [32,34], one evaluated sleep education [31], and two examined multicomponent digital interventions targeting loneliness or stress and well-being [30,35]. Comparators included treatment as usual [29], an attention control [33], waitlist or delayed-access conditions [30,32,35], minimal-contact or no-education controls [28,31], a non-MBCT comparison condition [34], and active comparisons between app features [36]. This diversity precludes interpreting the pooled estimates as the effect of a single interchangeable intervention. Instead, the random-effects estimates represent outcome-specific average effects across clinically diverse intervention models. The limited number of trials within each category prevented reliable comparisons between intervention classes. Outcome completion and attrition varied across studies, with differences in the extent and reporting of losses to follow-up (Supplementary Table S5). Intervention adherence and engagement were also heterogeneous, reflecting differences in intervention format, duration, adherence definitions, and reporting methods (Supplementary Table S6). Adverse-event ascertainment and deterioration reporting were limited or inconsistently reported across studies (Supplementary Table S7). Detailed study, intervention, comparator, and outcome characteristics are presented in Table 2.

### 3.4. Depressive Symptoms

**Figure 4:**
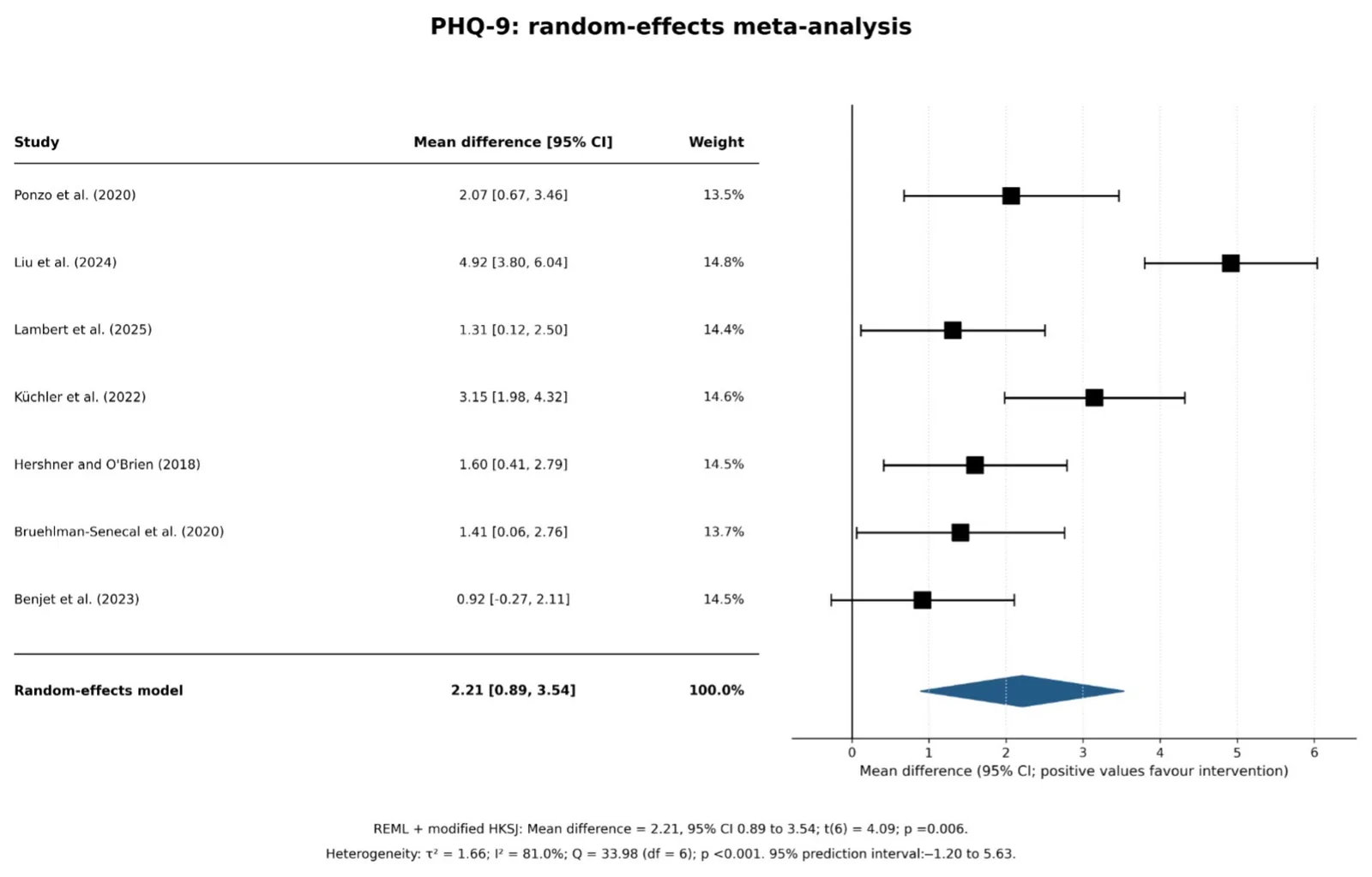
Random-effects meta-analysis of depressive symptoms measured with the PHQ-9 (7 RCTs; n = 2170). Positive mean differences favor the intervention. The REML model with modified Hartung–Knapp–Sidik–Jonkman inference yielded MD = 2.21 (95% CI, 0.89 to 3.54; t(6) = 4.09; *p* = 0.006), with τ^2^ = 1.66, I^2^ = 81.0%, Q(6) = 33.98 (*p* < 0.001), and a 95% prediction interval of −1.20 to 5.63 [29,30,31,32,33,34,35].

Seven RCTs contributed to the PHQ-9 meta-analysis. Under the REML random-effects model with modified HKSJ inference, mental health interventions reduced PHQ-9 scores by an average of 2.21 points relative to control conditions (MD = 2.21, 95% CI 0.89 to 3.54; t(6) = 4.09; *p* = 0.006). Heterogeneity was considerable (τ^2^ = 1.66; I^2^ = 81.0%; Q = 33.98, df = 6; *p* < 0.001). The 95% prediction interval ranged from −1.20 to 5.63 points and included no effect, indicating that the effect in a comparable future setting may differ from the estimated average (Figure 4).

### 3.5. Anxiety

**Figure 5:**
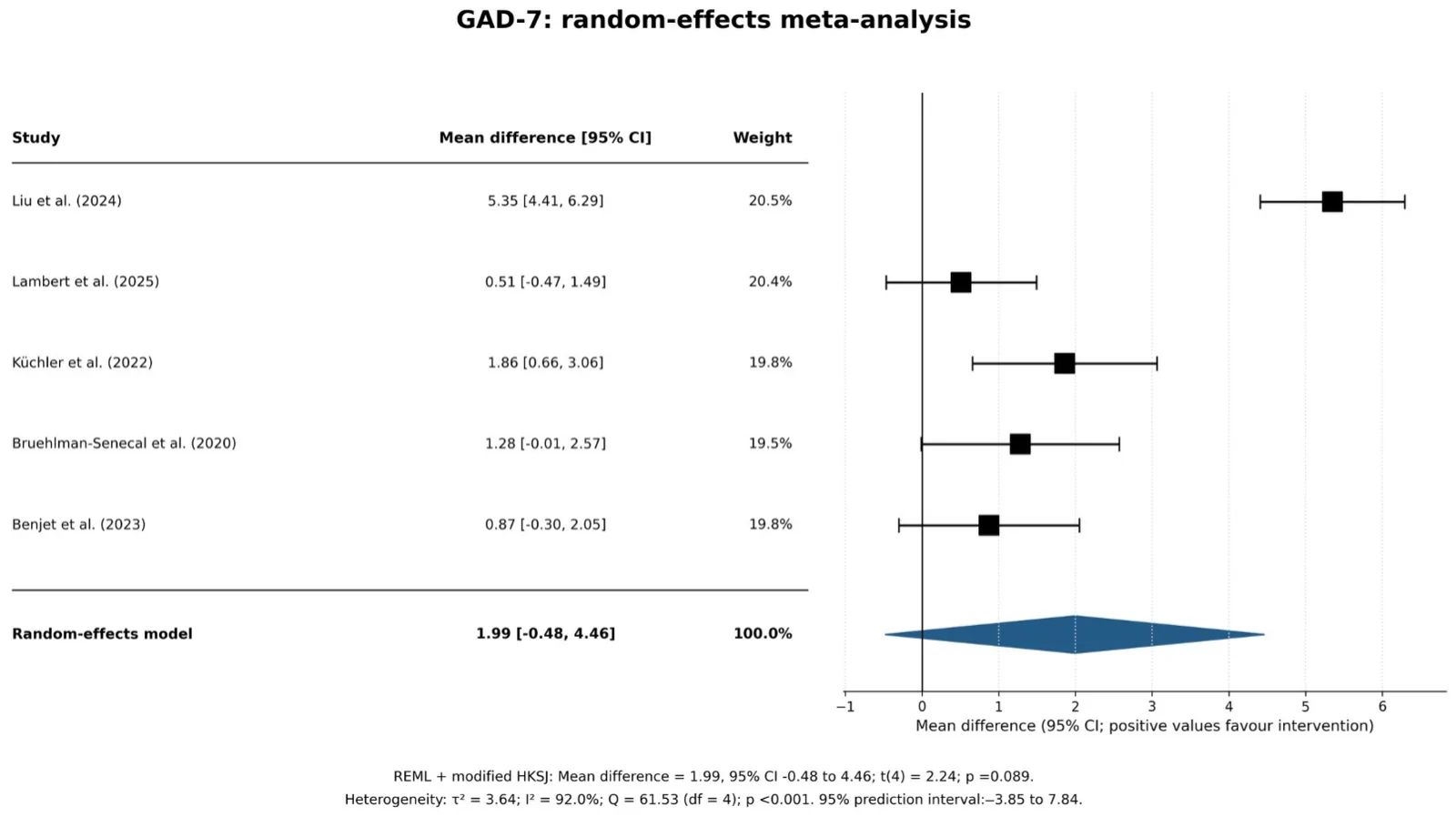
Random-effects meta-analysis of anxiety measured with the GAD-7 (5 RCTs; n = 1201). Positive mean differences favor the intervention. The REML model with modified Hartung–Knapp–Sidik–Jonkman inference yielded MD = 1.99 (95% CI, −0.48 to 4.46; t(4) = 2.24; *p* = 0.089), with τ^2^ = 3.64, I^2^ = 92.0%, Q(4) = 61.53 (*p* < 0.001), and a 95% prediction interval of −3.85 to 7.84 [29,30,32,33,34].

Five RCTs contributed to the GAD-7 meta-analysis. The pooled mean difference favored the interventions but was not statistically conclusive under modified HKSJ inference (MD = 1.99 points, 95% CI −0.48 to 4.46; t(4) = 2.24; *p* = 0.089). Heterogeneity was considerable (τ^2^ = 3.64; I^2^ = 92.0%; Q = 61.53, df = 4; *p* < 0.001), and the 95% prediction interval was wide and included no effect (−3.85 to 7.84). The available evidence therefore does not establish an average reduction in anxiety symptoms (Figure 5).

### 3.6. Sleep Quality

**Figure 6:**
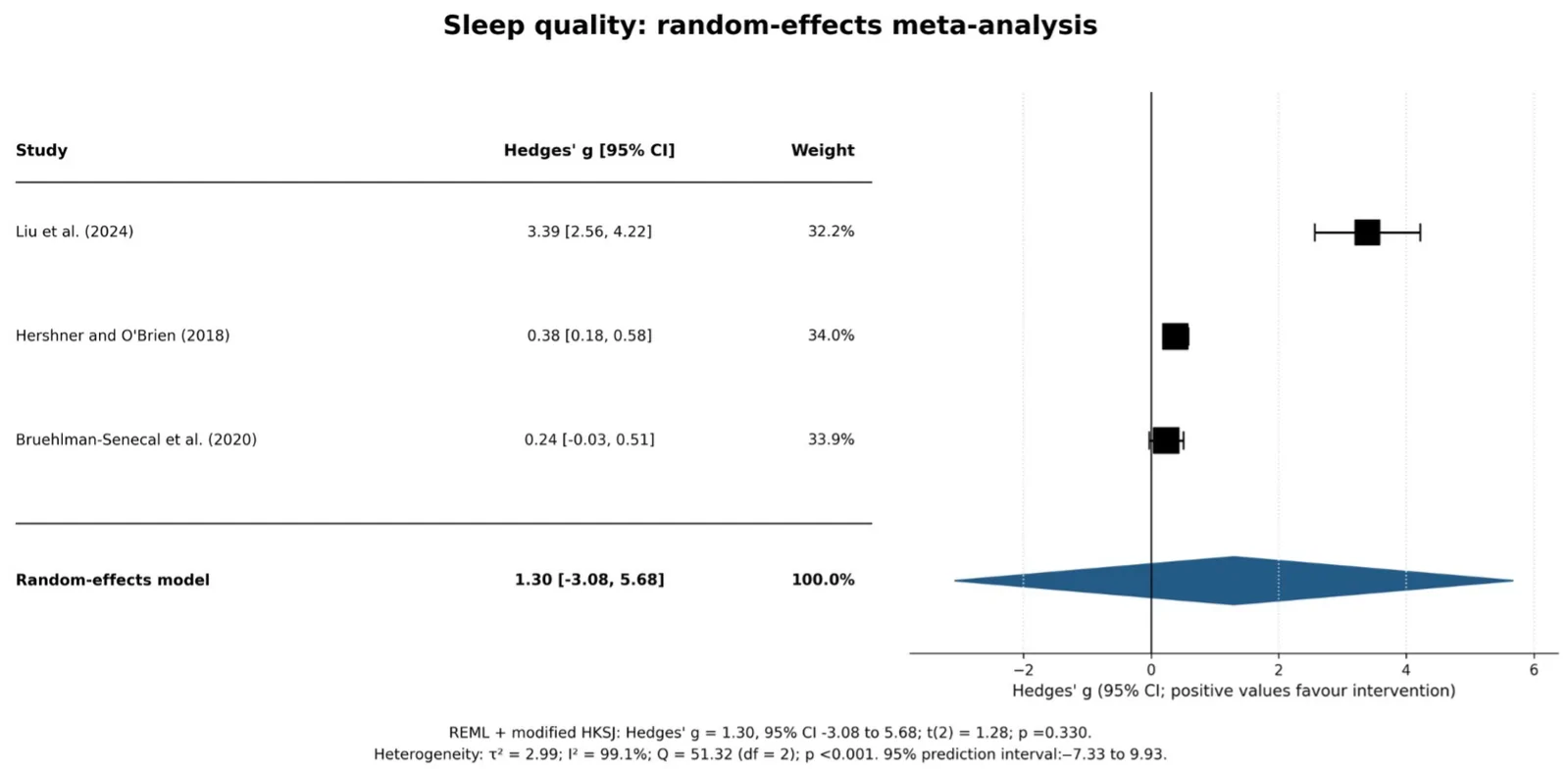
Random-effects meta-analysis of sleep quality (3 RCTs; n = 819). Effects were expressed as Hedges’ g because the contributing studies used non-equivalent PSQI scoring; positive values favor the intervention. The REML model with modified Hartung–Knapp–Sidik–Jonkman inference yielded g = 1.30 (95% CI, −3.08 to 5.68; t(2) = 1.28; *p* = 0.330), with τ^2^ = 2.99, I^2^ = 99.1%, Q(2) = 51.32 (*p* < 0.001), and a 95% prediction interval of −7.33 to 9.93 [30,31,34].

Three RCTs contributed to the sleep-quality meta-analysis. Because the studies reported non-equivalent PSQI scores, effects were standardized as Hedges’ g. The pooled effect was not statistically conclusive (g = 1.30, 95% CI −3.08 to 5.68; t(2) = 1.28; *p* = 0.330). Heterogeneity was considerable (τ^2^ = 2.99; I^2^ = 99.1%; Q = 51.32, df = 2; *p* < 0.001), and the 95% prediction interval ranged from −7.33 to 9.93. Because only three studies contributed, both the pooled estimate and prediction interval are highly uncertain. A same-scale audit identified only two studies reporting the global PSQI score; these were not pooled separately because the prespecified threshold of three studies was not met (Figure 6).

### 3.7. Sensitivity Analysis

**Figure 7:**
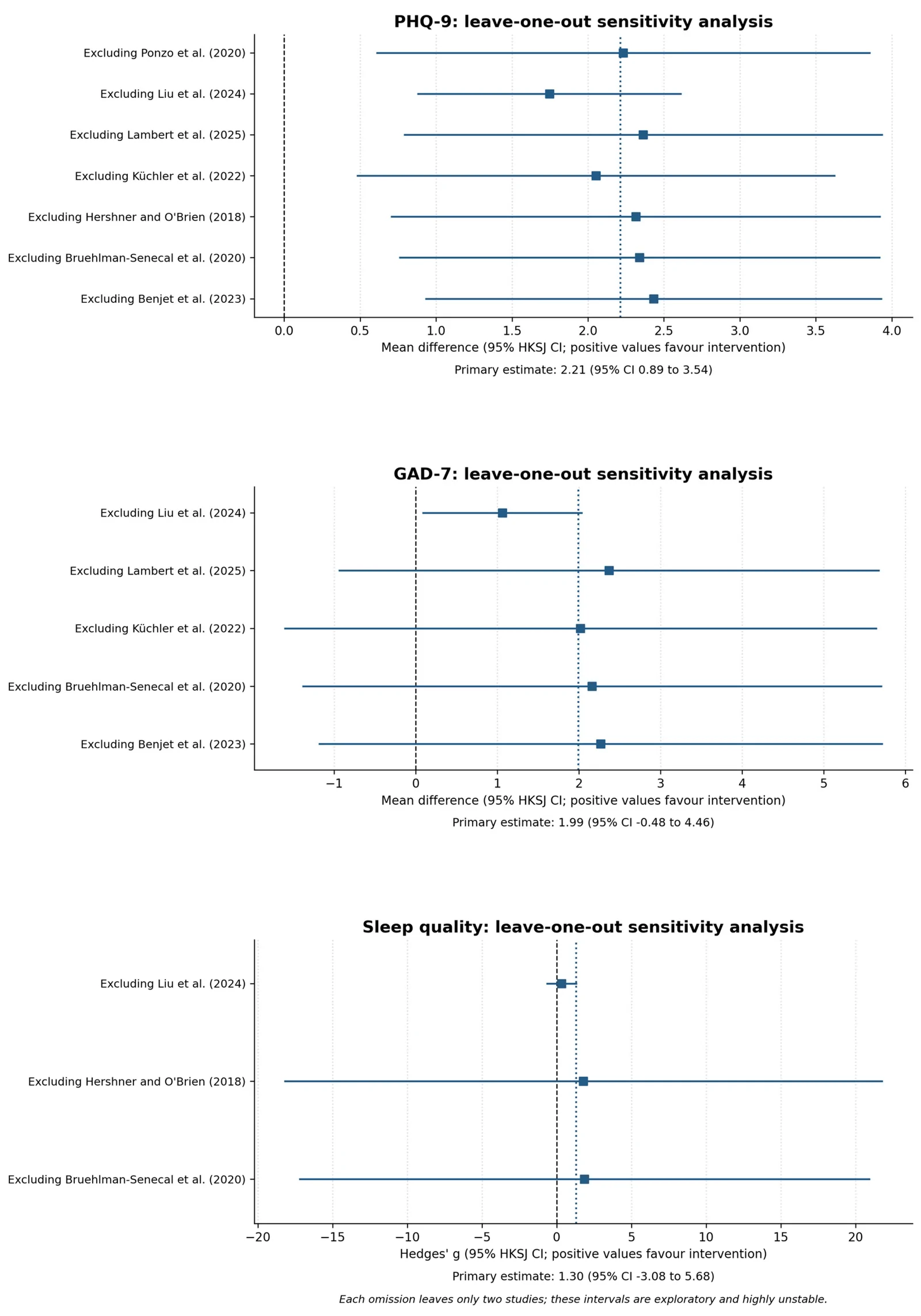
Leave-one-out sensitivity analyses for PHQ-9, GAD-7, and sleep-quality outcomes. Each square represents the REML pooled estimate after excluding the indicated study, and horizontal lines indicate modified HKSJ 95% confidence intervals. The dashed vertical line marks no effect, and the dotted line marks the primary pooled estimate. Each sleep-quality omission leaves only two studies; those intervals are exploratory and highly unstable [29,30,31,32,33,34,35].

Leave-one-out analyses showed that the pooled PHQ-9 effect remained statistically significant after each study was omitted, with mean differences ranging from 1.75 to 2.43 points. In contrast, the GAD-7 result was not statistically significant in four of the five analyses. Excluding Liu et al. substantially reduced heterogeneity (τ^2^ = 0.04; I^2^ = 10.2%) and produced a smaller estimate whose confidence interval narrowly excluded no effect (MD = 1.06, 95% CI 0.08 to 2.05). The anxiety result was therefore sensitive to this study. Each sleep-quality omission left only two studies, and all corresponding Hartung–Knapp confidence intervals included no effect. These two-study analyses were highly unstable and were considered exploratory. Complete numerical results are reported in Supplementary Table S8 and summarized in Figure 7.

### 3.8. Certainty of Evidence

Using GRADE, certainty was rated as low for depressive symptoms and very low for anxiety and sleep quality. Evidence for depressive symptoms was downgraded for risk of bias and inconsistency, whereas anxiety and sleep-quality evidence was additionally downgraded for imprecision. Indirectness was not considered serious for any outcome. No downgrade was applied for publication bias, although formal assessment was not possible because each synthesis included fewer than 10 studies. Outcome-specific judgments and explanations are presented in Table 3.

## 4. Discussion

### 4.1. Depressive Symptoms

The pooled PHQ-9 estimate favored the interventions; however, the 95% prediction interval included no effect, indicating that a comparable future university setting could observe little or no benefit. The substantial heterogeneity is clinically plausible because the trials included universal, selective, indicated, and treatment samples with different baseline severity and diagnostic status. Interventions also differed in theoretical basis, human guidance, intensity, duration, adherence, comparator conditions, assessment timing, and analysis population. Together with missing outcome data and unblinded self-report in several trials, this diversity explains the low certainty rating and limits generalizability. The pooled estimate should therefore be interpreted as an uncertain average across heterogeneous programs rather than an effect expected in every university population. Comparable variability across intervention models and populations has been reported previously [2,18,19].

Within the university context, digital and self-guided interventions are especially relevant, as they can assist in supporting large student populations. Previous reviews found small to moderate effects of these strategies on symptoms of depression [37,38]. Individual RCTs of internet-based programs, mobile therapies, Acceptance and Commitment Therapy and mindfulness-based approaches also found reductions in depressive symptoms [34,39,40,41,42]. But the wide range in the current analysis suggests that the level of benefit is unlikely to be consistent across student demographics or programs.

### 4.2. Anxiety

A pooled analysis found mental health interventions to be associated with lower anxiety symptoms, but the considerable between-study heterogeneity makes the magnitude of this effect difficult to interpret. Similar variability has been reported in previous reviews of university populations, with interventions generally reducing anxiety, but with differing effects across studies [21,43]. Internet-delivered cognitive behavioral therapy was demonstrated to be effective in anxiety and digital interventions may also be useful in tackling both anxiety and depressive symptoms in university students [21,43]. Mindfulness-based approaches have also been linked to decreases in anxiety symptoms [44].

Cognitive behavioral and mindfulness-based interventions should not be regarded as interchangeable. CBT-oriented programs target maladaptive cognitions and avoidance through structured strategies such as cognitive restructuring, behavioral activation, problem solving, and exposure, whereas mindfulness-based approaches emphasize present-moment awareness and reduced reactivity to rumination and distress [21,43,44]. These mechanisms may produce different outcome profiles: CBT and CBT-I are more directly aligned with symptom-specific cognitive and behavioral patterns, whereas mindfulness may exert broader transdiagnostic effects [23]. Nevertheless, some trials found little or no advantage over their comparator conditions [45], and the included studies differed in guidance, intensity, adherence, delivery mode, baseline severity, and comparator type. Because few studies represented each approach and no reliable head-to-head or subgroup analysis was possible, the present evidence does not establish the superiority of CBT or mindfulness. Apparent differences may reflect study design and population characteristics rather than intervention type alone.

### 4.3. Sleep Quality

The pooled analysis suggests that mental health interventions may improve sleep quality among university students, but this finding is less robust than those observed for depressive and anxiety symptoms. Only three studies contributed to the analysis, heterogeneity was substantial, and the pooled effect lost statistical significance when individual studies were removed. The magnitude of the effect should therefore be interpreted cautiously. Previous research nevertheless points in a similar direction. A recent systematic review and meta-analysis found that psychological interventions improved sleep quality in university students, with greater effects being observed for cognitive behavioral therapy for insomnia (CBT-I) than mindfulness-based approaches [23].

Another interpretation of these results is a close relation between sleep and depression. Non-pharmacological interventions have been linked to improved sleep quality and reduced depression in individuals with depression [24]. Furthermore, the relationship could be bi-directional, as the treatment of sleep disturbances was associated with an improvement in depressive symptoms [46]. These results together advocate for the close interplay between sleep and depression. However, they do not show whether improvement in one of the outcomes is directly associated with improvement in the other.

The results may also be affected by the design of the interventions and control conditions. For instance, recent trials in university students have reported smaller differences when interventions were compared with active digital controls [47]. Thus, variation across studies may be due to adherence, intervention content and choice of comparator. Given the small evidence base and very low certainty of evidence for sleep quality in the present review, firm conclusions cannot be drawn at this time about the magnitude or durability of the effect. Further RCTs are required, particularly among students with clinically significant depressive symptoms, using standardized sleep measures and longer follow-up periods.

### 4.4. Limitations and Strengths

Several limitations should be considered. Differences in populations, baseline severity, intervention content, guidance, intensity, adherence, comparators, and assessment timing reduced study comparability and contributed to heterogeneity. Confidence was also limited by the missing outcome data and unblinded self-reported assessments. Although pooling was restricted to compatible instruments, studies used the PHQ-8 or PHQ-9 and non-equivalent PSQI scores, constraining the number of eligible comparisons. These measures reflect symptoms rather than clinical diagnoses or objective sleep characteristics.

The analyses used immediate post-intervention means, which do not establish durability. Alternative approaches using change scores, adjusted estimates, or different estimators of between-study variance could produce different results. Moreover, the small number of studies made heterogeneity and prediction intervals uncertain and precluded reliable subgroup analyses, meta-regression, and formal assessment of small-study effects.

Strengths include PROSPERO registration, predefined PICOS criteria, coverage of seven information sources, restriction to RCTs, and outcome specific RoB 2 and GRADE assessments. Instrument-specific pooling, modified Hartung–Knapp inference, prediction intervals, and sensitivity analyses supported cautious interpretation. Future RCTs should use standardized outcomes, intention-to-treat analyses, transparent reporting of adherence and adverse events, and longer follow-up.

### 4.5. Implications for Practice and Research

Although previous reviews suggest that psychological and sleep-focused interventions may benefit university students [19,22,23], the present findings do not support recommending a specific intervention or widespread implementation. The average effect on depressive symptoms was favorable, but its transferability remains uncertain; effects on anxiety and sleep quality were inconclusive. Universities introducing digital, cognitive behavioral, mindfulness-based, or sleep-focused programs should therefore evaluate uptake, adherence, attrition, adverse events, and outcomes within their own services.

Decisions concerning routine screening or resource allocation should rely on independent epidemiological evidence and local needs [48], rather than on these pooled intervention estimates alone. Future RCTs should use clinically well-characterized samples, comparable outcomes and assessment times, intention-to-treat analyses, and longer follow-up, while examining whether guidance, intensity, baseline severity, or intervention type modify treatment effects.

## 5. Conclusions

Mental health interventions may reduce depressive symptoms among university students on average, but considerable heterogeneity and prediction intervals that include no effect limit the generalizability of this finding. The pooled evidence for anxiety and sleep quality was inconclusive after applying methods that account for uncertainty from the small number of studies. Risk-of-bias concerns, heterogeneous interventions and populations, differing analysis sets, and the low to very low certainty of the evidence preclude firm clinical or public health recommendations. Further well-designed randomized controlled trials using standardized outcome definitions, transparent intention-to-treat analyses, and longer follow-up are required.

## Figures and Tables

**Table 1:** Selection criteria used in the systematic review and meta-analysis.

Category	Inclusion Criteria	Exclusion Criteria
Population	Adults aged ≥18 years currently enrolled in undergraduate or postgraduate study at a university or college. Universal, selective, indicated, and clinically diagnosed samples were eligible. Neither elevated depressive symptoms nor a formal diagnosis of depression was required.	Participants aged <18 years; non-student populations; mixed samples when data for university students could not be extracted separately; samples recruited primarily because of a neurological or medical condition unrelated to the intervention target.
Intervention	Any structured, non-pharmacological intervention with an explicit mental health or sleep-related preventive or therapeutic aim, including psychological, behavioral, psychoeducational, mindfulness-based, problem-solving, digitally delivered, and sleep-focused interventions. No minimum intervention duration or number of sessions was required.	Pharmacological interventions alone; interventions without an explicit mental health or sleep-related component; or studies in which the intervention was used only as an assessment condition.
Comparison	Any randomized active or inactive comparator, including usual care, waitlist or delayed access, minimal contact, no intervention, psychoeducation, or an alternative digital intervention.	Absence of a concurrent randomized comparator.
Outcomes	Mandatory: depressive symptoms assessed with a validated instrument at baseline and at least one post-randomization time point. Secondary: anxiety symptoms and sleep quality when co-reported. Availability of data suitable for meta-analysis was not required for inclusion in the qualitative synthesis.	No depressive-symptom outcome. Studies reporting only anxiety or sleep outcomes were not eligible.
Study design	Randomized controlled trials.	Controlled, cross-sectional, retrospective and prospective, non-randomized studies.

**Table 2:** Characteristics of the randomized controlled trials included in the systematic review.

Study	Country	Participants and Clinical Scope	Sample and Allocation	Mean Age, Years	Intervention	Comparator	Dose and Assessment	Outcomes
[28]	United States	Student military veterans with depressive symptoms (indicated)	N = 24; ePST, n = 12; MCC, n = 12	32.7	Computer-guided problem-solving treatment (ePST)	Minimal-contact control	Six weekly sessions; session duration NR; PHQ-9 assessed weekly through week 6 and ISI at posttreatment	D: PHQ-9 A: NR S: ISI
[29]	Colombia and Mexico	Undergraduates with GAD-7 and/or PHQ-9 scores of 10 or higher (indicated)	N = 1319; guided i-CBT, n = 445; self-guided i-CBT, n = 439; TAU, n = 435	21.4	Culturally adapted transdiagnostic i-CBT, delivered with or without guidance	Mental health services routinely available at each university (TAU)	Seven core modules designed for 8 weeks; self-paced; weekly feedback in the guided arm; assessment at 3 months	D: PHQ-9 A: GAD-7, PHQ-ADS S: NR
[30]	United States	Incoming first-year students without a symptom threshold (universal)	N = 221; Nod, n = 100; delayed-access control, n = 121	18.7	Self-guided Nod smartphone app addressing loneliness, social connection, and self-compassion	Delayed access to Nod after 4 weeks	Four weeks of self-directed access; frequency and session duration not prescribed; assessments at weeks 2, 4, and 8	D: PHQ-9 A: GAD-7 S: PSQI subjective sleep-quality item
[31]	United States	Students aged 18 years or older at a public Midwestern university (universal)	Baseline sample N = 549; sleep education, n = 254; no education, n = 295	21.9	Personalized Sleep to Stay Awake online sleep-education module	No sleep education	Single approximately 20 min module; assessments at 1 and 8 weeks	D: PHQ-9 A: NR S: PSQI, ESS, SHI
[32]	Germany, Switzerland, and Austria	College students with low-to-moderate mindfulness (FMI < 37; selective)	N = 150 enrolled; ITT sample N = 149 (StudiCare, n = 74; waitlist, n = 75)	28.25	Guided StudiCare Mindfulness combining ACT, MBSR, and stress-management content	Waitlist plus support-information leaflet; unrestricted access to TAU	Five weekly modules (approximately 60 min each) plus daily mindfulness practice; assessment at 6 weeks	D: PHQ-9 A: GAD-7 S: NR
[33]	United Kingdom	Undergraduate and postgraduate students without a symptom threshold (universal)	N = 468 randomized; COMET, n = 239; attention control, n = 229	22.49	Web-based COMET single-session intervention: behavioral activation, cognitive restructuring, gratitude, and self-compassion	Attention-control questionnaire battery	One 60–75 min session; follow-up assessments at 2 and 4 weeks	D: PHQ-9 A: GAD-7 S: NR
[34]	China	Undergraduates with psychiatrist-confirmed, first-episode untreated MDD (treatment)	N = 60 randomized (MBCT, n = 30; waitlist, n = 30); analyzed n = 26 and n = 30, respectively	Mean NR; range 19–29	Manualized mindfulness-based cognitive therapy (MBCT)	Waitlist, with MBCT offered after the waiting period	Eight weekly 150 min sessions plus one full practice day; assessments at weeks 4 and 8	D: PHQ-9 A: GAD-7 S: global PSQI
[35]	United Kingdom	Full-time students aged 18–25 years with elevated anxiety or stress on the DASS-21 (indicated)	N = 262 randomized (BioBase, n = 130; waitlist, n = 132); baseline analysis N = 146 (n = 72; n = 74)	19.9	BioBase mobile app plus BioBeam wearable: psychoeducation, mood tracking, and in-the-moment exercises	Waitlist; access to the app and wearable after 8 weeks	Four weeks; daily use for at least 5 min encouraged; assessments at weeks 2, 4, and 6	D: PHQ-9, DASS-21-D A: STAI-S-6, DASS-21-A S: NR
[36]	United States	College students aged 18–30 years with and without depressive symptoms (universal)	N = 209 across four active factorial conditions; arm-level n NR in the main report	19.97	All groups received the AirHeart bCBT app; virtual-agent conversation and animation were varied in a 2 × 2 factorial design	Alternative active app variants; no inactive control	Two weeks; use every other day with at least eight uses for full completion; assessment at 2 weeks	D: PHQ-8 A: NR S: NR

A: anxiety; ACT: acceptance and commitment therapy; bCBT: brief cognitive behavioral therapy; COMET: Competitive Memory Training; D: depression; DASS-21-A: Depression Anxiety Stress Scales-21 anxiety subscale; DASS-21-D: Depression Anxiety Stress Scales-21 depression subscale; ESS: Epworth Sleepiness Scale; FMI: Freiburg Mindfulness Inventory; GAD-7: Generalized Anxiety Disorder-7; i-CBT: internet-delivered cognitive behavioral therapy; ISI: Insomnia Severity Index; ITT: intention to treat; MBCT: mindfulness-based cognitive therapy; MBSR: mindfulness-based stress reduction; MCC: minimal-contact control; MDD: major depressive disorder; NR: not reported; PHQ-8: Patient Health Questionnaire-8; PHQ-9: Patient Health Questionnaire-9; PHQ-ADS: Patient Health Questionnaire Anxiety and Depression Scale; PSQI: Pittsburgh Sleep Quality Index; S: sleep; SHI: Sleep Hygiene Index; STAI-S-6: six-item State-Trait Anxiety Inventory-State; TAU: treatment as usual.

**Table 3:** Evaluation of methodological quality using the GRADEpro tool.

Outcome	Studies and Participants	Risk of Bias	Inconsistency	Indirectness	Imprecision	Publication Bias	Pooled Effect	Certainty
Depressive symptoms PHQ-9 2 weeks to 3 months	7 RCTs n = 2170 ^a^	Serious ^c^	Serious ^d^	Not serious	Not serious	Not downgraded ^k^	MD = 2.21 favoring intervention (95% CI 0.89 to 3.54; 95% PI −1.20 to 5.63), equivalent to a 2.21-point lower mean PHQ-9 score ^b^	Low
Anxiety symptoms GAD-7 2 weeks to 3 months	5 RCTs n = 1201 ^a^	Serious ^e^	Serious ^f^	Not serious	Serious ^g^	Not downgraded ^k^	MD = 1.99 favoring intervention (95% CI −0.48 to 4.46; 95% PI −3.85 to 7.84), equivalent to a 1.99-point lower mean GAD-7 score ^b^	Very low
Sleep quality PSQI-based 4 to 8 weeks	3 RCTs n = 819 ^a^	Serious ^h^	Serious ^i^	Not serious	Serious ^j^	Not downgraded ^k^	Hedges’ g = 1.30 favoring intervention (95% CI −3.08 to 5.68; 95% PI −7.33 to 9.93); an absolute PSQI-point difference was not estimable ^b^	Very low

^a^ Total n represents the analyzed sample; the reported model sample was used when time-point-specific data were unavailable. ^b^ Positive estimates favor the intervention; lower PHQ-9, GAD-7, and PSQI scores indicate improvement. ^c^ One-level downgrade for risk of bias: two PHQ-9 trials were at high risk, four had some concerns, and one was at low risk, mainly due to missing data and unblinded self-report. ^d^ One-level downgrade for inconsistency: I^2^ = 81.0%, τ^2^ = 1.66, and the 95% PI crossed the null. ^e^ One-level downgrade for risk of bias: one GAD-7 trial was at high risk, three had some concerns, and one was at low risk. ^f^ One-level downgrade for inconsistency: I^2^ = 92.0%, τ^2^ = 3.64; the 95% PI crossed the null, and leave-one-out results were sensitive to Liu et al. ^g^ One-level downgrade for imprecision: the 95% CI crossed the null and included both possible harm and clinically relevant benefit. ^h^ One-level downgrade for risk of bias: two sleep-quality trials had some concerns, and one was at low risk, mainly due to unblinded self-report. ^i^ One-level downgrade for inconsistency: I^2^ = 99.1%, τ^2^ = 2.99; effect sizes and PSQI scoring differed substantially, and the 95% PI crossed the null. ^j^ One-level downgrade for imprecision: only three trials contributed, and the 95% CI included large effects in both directions. ^k^ Publication bias was not downgraded because formal assessment was not feasible with fewer than 10 studies per outcome. CI: confidence interval; GAD-7: Generalized Anxiety Disorder-7; GRADE: Grading of Recommendations Assessment: Development and Evaluation; MD: mean difference; PHQ-9: Patient Health Questionnaire-9; PI: prediction interval; PSQI: Pittsburgh Sleep Quality Index; RCT: randomized controlled trial.

## Data Availability

The authors confirm that the data supporting the findings of this study are available within the article.

## Supplementary Materials

The following supporting information can be downloaded at: https://www.mdpi.com/article/10.3390/jcm15197630/s1, Table S1 checklist 2020 [49]; Table S2: Database-specific search strategies; Table S3: Domain-level Cochrane Risk of Bias 2 (RoB 2) judgments for included studies; Table S4: Supporting rationales for domain-level Cochrane Risk of Bias 2 (RoB 2) judgments; Table S5: Outcome completion and attrition at the study level; Table S6: Intervention adherence and engagement at the study level; Table S7: Adverse-event and deterioration reporting at the study level; Table S8: Leave-one-out sensitivity analyses for depressive symptoms, anxiety, and sleep quality.
