# Supplementary material for: Effects of Mental Health Interventions on Depressive Symptoms, Anxiety, and Sleep Quality in University Students: A Systematic Review and Meta-Analysis
Source: J Clin Med. 2026 Oct 1;15(19):7630. doi: 10.3390/jcm15197630 (PMC13665775; doi:10.3390/jcm15197630)
Supplement: Supplementary file 1 [file jcm-15-07630-s001.zip › jcm-4550527-supplementary.pdf]

Supplementary Table S1: PRISMA Checklist 2020

SECTION	ITEM	PRISMA-ScR CHECKLIST ITEM	REPORTED ON PAGE #
**TITLE**			
Title	1	Identify the report as a scoping review.	p. 1
**ABSTRACT**			
Structured summary	2	Provide a structured summary that includes (as applicable): background, objectives, eligibility criteria, sources of evidence, charting methods, results, and conclusions that relate to the review questions and objectives.	pp. 1-2
**INTRODUCTION**			
Rationale	3	Describe the rationale for the review in the context of what is already known. Explain why the review questions/objectives lend themselves to a scoping review approach.	pp. 2-3
Objectives	4	Provide an explicit statement of the questions and objectives being addressed with reference to their key elements (e.g., population or participants, concepts, and context) or other relevant key elements used to conceptualize the review questions and/or objectives.	p. 3
**METHODS**			
Protocol and registration	5	Indicate whether a review protocol exists; state if and where it can be accessed (e.g., a Web address); and if available, provide registration information, including the registration number.	p. 3
Eligibility criteria	6	Specify characteristics of the sources of evidence used as eligibility criteria (e.g., years considered, language, and publication status), and provide a rationale.	pp. 3-4. Table 1
Information sources*	7	Describe all information sources in the search (e.g., databases with dates of coverage and contact with authors to identify additional sources), as well as the date the most recent search was executed.	p. 4
Search	8	Present the full electronic search strategy for at least 1 database, including any limits used, such that it could be repeated.	p. 4
Selection of sources of evidence†	9	State the process for selecting sources of evidence (i.e., screening and eligibility) included in the scoping review.	p. 4
Data charting process‡	10	Describe the methods of charting data from the included sources of evidence (e.g., calibrated forms or forms that have been tested by the team before their use, and whether data charting was done independently or in duplicate) and any processes for obtaining and confirming data from investigators.	p. 4
Data items	11	List and define all variables for which data were sought and any assumptions and simplifications made.	pp. 3-5
Critical appraisal of individual sources of evidence§	12	If done, provide a rationale for conducting a critical appraisal of included sources of evidence; describe the methods used and how this information was used in any data synthesis (if appropriate).	p. 5
Synthesis of results	13	Describe the methods of handling and summarizing the data that were charted.	p. 5, Figure 1
**RESULTS**			
Selection of sources of evidence	14	Give numbers of sources of evidence screened, assessed for eligibility, and included in the review, with reasons for exclusions at each stage, ideally using a flow diagram.	p. 5
Characteristics of sources of evidence	15	For each source of evidence, present characteristics for which data were charted and provide the citations.	pp. 7-11, Table 2

SECTION	ITEM	PRISMA-ScR CHECKLIST ITEM	REPORTED ON PAGE #
Critical appraisal within sources of evidence	16	If done, present data on critical appraisal of included sources of evidence (see item 12).	p. 6, Figures 2-3
Results of individual sources of evidence	17	For each included source of evidence, present the relevant data that were charted that relate to the review questions and objectives.	pp. 7-16, Table 2, Figures 4-6
Synthesis of results	18	Summarize and/or present the charting results as they relate to the review questions and objectives.	pp. 12-14, 17-20
**DISCUSSION**			
Summary of evidence	19	Summarize the main results (including an overview of concepts, themes, and types of evidence available), link to the review questions and objectives, and consider the relevance to key groups.	pp. 20-22
Limitations	20	Discuss the limitations of the scoping review process.	p. 22
Conclusions	21	Provide a general interpretation of the results with respect to the review questions and objectives, as well as potential implications and/or next steps.	pp. 22-24
**FUNDING**			
Funding	22	Describe sources of funding for the included sources of evidence, as well as sources of funding for the scoping review. Describe the role of the funders of the scoping review.	p. 24

JBI = Joanna Briggs Institute; PRISMA-ScR = Preferred Reporting Items for Systematic reviews and Meta-Analyses extension for Scoping Reviews.
* Where *sources of evidence* (see second footnote) are compiled from, such as bibliographic databases, social media platforms, and Web sites.
† A more inclusive/heterogeneous term used to account for the different types of evidence or data sources (e.g., quantitative and/or qualitative research, expert opinion, and policy documents) that may be eligible in a scoping review as opposed to only studies. This is not to be confused with *information sources* (see first footnote).
‡ The frameworks by Arksey and O'Malley (6) and Levac and colleagues (7) and the JBI guidance (4, 5) refer to the process of data extraction in a scoping review as data charting.
§ The process of systematically examining research evidence to assess its validity, results, and relevance before using it to inform a decision. This term is used for items 12 and 19 instead of "risk of bias" (which is more applicable to systematic reviews of interventions) to include and acknowledge the various sources of evidence that may be used in a scoping review (e.g., quantitative and/or qualitative research, expert opinion, and policy document).

From: Tricco AC, Lillie E, Zarin W, O'Brien KK, Colquhoun H, Levac D, et al. PRISMA Extension for Scoping Reviews (PRISMAScR): Checklist and Explanation. Ann Intern Med. 2018;169:467–473. doi: 10.7326/M18-0850

Supplementary Table S2. Database-specific search strategies.

**MEDLINE (via PubMed) complete search strategy**
("university student"[Title/Abstract] OR "university students"[Title/Abstract] OR "college student"[Title/Abstract] OR "college students"[Title/Abstract] OR undergraduate*[Title/Abstract] OR "graduate student"[Title/Abstract] OR "graduate students"[Title/Abstract] OR postgraduate*[Title/Abstract] OR "higher education student"[Title/Abstract] OR "higher education students"[Title/Abstract] OR (tertiary[Title/Abstract] AND education[Title/Abstract] AND (student[Title/Abstract] OR students[Title/Abstract]))) AND ("Depression"[Mesh] OR "Depressive Disorder"[Mesh] OR "Anxiety"[Mesh] OR "Anxiety Disorders"[Mesh] OR "Sleep Wake Disorders"[Mesh] OR depress*[Title/Abstract] OR anxi*[Title/Abstract] OR insomnia*[Title/Abstract] OR "sleep quality"[Title/Abstract] OR "sleep disturbance"[Title/Abstract] OR "sleep disturbances"[Title/Abstract] OR "sleep problem"[Title/Abstract] OR "sleep problems"[Title/Abstract] OR "sleep disorder"[Title/Abstract] OR "sleep disorders"[Title/Abstract] OR "psychological distress"[Title/Abstract]) AND ("Psychotherapy"[Mesh] OR "Cognitive Behavioral Therapy"[Mesh] OR "Mindfulness"[Mesh] OR intervention*[Title/Abstract] OR treatment*[Title/Abstract] OR therap*[Title/Abstract] OR psychotherap*[Title/Abstract] OR (cognitive[Title/Abstract] AND behavio*[Title/Abstract] AND therap*[Title/Abstract]) OR mindfulness[Title/Abstract] OR psychoeducat*[Title/Abstract] OR "problem solving"[Title/Abstract] OR (digital[Title/Abstract] AND intervention*[Title/Abstract]) OR (internet-based[Title/Abstract] AND intervention*[Title/Abstract]) OR (web-based[Title/Abstract] AND intervention*[Title/Abstract]) OR (mobile[Title/Abstract] AND intervention*[Title/Abstract]) OR (mobile[Title/Abstract] AND (app[Title/Abstract] OR apps[Title/Abstract] OR application*[Title/Abstract])) OR (smartphone[Title/Abstract] AND (app[Title/Abstract] OR apps[Title/Abstract] OR application*[Title/Abstract]))) AND (randomized controlled trial[Publication Type] OR controlled clinical trial[Publication Type] OR random*[Title/Abstract] OR trial[Title/Abstract] OR placebo[Title/Abstract] OR waitlist[Title/Abstract] OR "wait-list"[Title/Abstract]) NOT (animals[MeSH Terms] NOT humans[MeSH Terms]) NOT (review[Publication Type] OR systematic review[Publication Type] OR meta-analysis[Publication Type])
Search details: Last search date: 30 July 2026<br>Original yield: n = 910<br>Language: no filter<br>Publication date: no restriction<br>Other limits in syntax: animal-only studies and review publication types excluded

**Scopus**<br>**(Elsevier) complete search strategy**
TITLE-ABS-KEY(<br>("university student*" OR "college student*" OR undergraduate* OR "graduate student*" OR postgraduate* OR "higher education student*" OR "tertiary education student*")<br>AND (depress* OR anxi* OR insomnia* OR "sleep quality" OR "sleep disturbance*" OR "sleep problem*" OR "sleep disorder*" OR "psychological distress")<br>AND (intervention* OR treatment* OR therap* OR psychotherap* OR "cognitive behavioral therap*" OR "cognitive behavioural therap*" OR mindfulness OR psychoeducat* OR "problem solving" OR "digital intervention*" OR "internet-based intervention*" OR "web-based intervention*" OR "mobile intervention*" OR "mobile app*" OR "smartphone app*")<br>AND (random* OR trial OR placebo OR waitlist OR "wait-list")<br>)
Search details: Last search date: 30 July 2026<br>Original yield: n = 1,552<br>Language: no filter<br>Publication date: no restriction<br>Document type: no database filter; eligibility assessed during screening

**Cochrane Central Register of Controlled Trials (CENTRAL)**<br>**(via Cochrane Library) complete search strategy**
#1 ("university student*" OR "college student*" OR undergraduate* OR "graduate student*" OR postgraduate* OR "higher education student*" OR "tertiary education student*"):ti,ab,kw #2 (depress* OR anxi* OR insomnia* OR "sleep quality" OR "sleep disturbance*" OR "sleep problem*" OR "sleep disorder*" OR "psychological distress"):ti,ab,kw #3 (intervention* OR treatment* OR therap* OR psychotherap* OR "cognitive behavioral therap*" OR "cognitive behavioural therap*" OR mindfulness OR psychoeducat* OR "problem solving" OR "digital intervention*" OR "internet-based intervention*" OR "web-based intervention*" OR "mobile intervention*" OR "mobile app*" OR "smartphone app*"):ti,ab,kw #4 #1 AND #2 AND #3<br>Search source: Trials (CENTRAL). No separate randomized-trial filter was applied because CENTRAL is a controlled-trials database.
Search details: Search period: 30 July 2026<br>Original yield: n = 243<br>Search source: Trials<br>Language: no filter<br>Publication date: no restriction

**Web of Science Core Collection<br>(Clarivate) complete search strategy**
TS=("university student*" OR "college student*" OR undergraduate* OR "graduate student*" OR postgraduate* OR "higher education student*" OR "tertiary education student*")<br>AND TS=(depress* OR anxi* OR insomnia* OR "sleep quality" OR "sleep disturbance*" OR "sleep problem*" OR "sleep disorder*" OR "psychological distress")<br>AND TS=(intervention* OR treatment* OR therap* OR psychotherap* OR "cognitive behavioral therap*" OR "cognitive behavioural therap*" OR mindfulness OR psychoeducat* OR "problem solving" OR "digital intervention*" OR "internet-based intervention*" OR "web-based intervention*" OR "mobile intervention*" OR "mobile app*" OR "smartphone app*")<br>AND TS=(random* OR trial OR placebo OR waitlist OR "wait-list")
Search details: Last search date: 30 July 2026<br>Original yield: n = 1,521<br>Language: no filter<br>Timespan: all years<br>Document type: no database filter; eligibility assessed during screening

**APA PsycINFO<br>(via EBSCOhost) complete search strategy**
S1 TI ("university student*" OR "college student*" OR undergraduate* OR "graduate student*" OR postgraduate* OR "higher education student*" OR "tertiary education student*")<br>S2 AB ("university student*" OR "college student*" OR undergraduate* OR "graduate student*" OR postgraduate* OR "higher education student*" OR "tertiary education student*")<br>S3 S1 OR S2<br>S4 TI (depress* OR anxi* OR insomnia* OR "sleep quality" OR "sleep disturbance*" OR "sleep problem*" OR "sleep disorder*" OR "psychological distress")<br>S5 AB (depress* OR anxi* OR insomnia* OR "sleep quality" OR "sleep disturbance*" OR "sleep problem*" OR "sleep disorder*" OR "psychological distress")<br>S6 S4 OR S5<br>S7 TI (intervention* OR treatment* OR therap* OR psychotherap* OR "cognitive behavioral therap*" OR "cognitive behavioural therap*" OR mindfulness OR psychoeducat* OR "problem solving" OR "digital intervention*" OR "internet-based intervention*" OR "web-based intervention*" OR "mobile intervention*" OR "mobile app*" OR "smartphone app*")<br>S8 AB (intervention* OR treatment* OR therap* OR psychotherap* OR "cognitive behavioral therap*" OR "cognitive behavioural therap*" OR mindfulness OR psychoeducat* OR "problem solving" OR "digital intervention*" OR "internet-based intervention*" OR "web-based intervention*" OR "mobile intervention*" OR "mobile app*" OR "smartphone app*")<br>S9 S7 OR S8<br>S10 TI (random* OR trial OR placebo OR waitlist OR "wait-list")<br>S11 AB (random* OR trial OR placebo OR waitlist OR "wait-list")<br>S12 S10 OR S11<br>S13 S3 AND S6 AND S9 AND S12
Search details: Last search date: 30 July 2026<br>Original yield: n = 388<br>Language: no filter<br>Publication date: no restriction<br>Publication type: no database filter; eligibility assessed during screening

**CINAHL Complete**<br>**(via EBSCOhost) complete search strategy**
S1 TI ("university student*" OR "college student*" OR undergraduate* OR "graduate student*" OR postgraduate* OR "higher education student*" OR "tertiary education student*")<br>S2 AB ("university student*" OR "college student*" OR undergraduate* OR "graduate student*" OR postgraduate* OR "higher education student*" OR "tertiary education student*")<br>S3 S1 OR S2<br>S4 TI (depress* OR anxi* OR insomnia* OR "sleep quality" OR "sleep disturbance*" OR "sleep problem*" OR "sleep disorder*" OR "psychological distress")<br>S5 AB (depress* OR anxi* OR insomnia* OR "sleep quality" OR "sleep disturbance*" OR "sleep problem*" OR "sleep disorder*" OR "psychological distress")<br>S6 S4 OR S5<br>S7 TI (intervention* OR treatment* OR therap* OR psychotherap* OR "cognitive behavioral therap*" OR "cognitive behavioural therap*" OR mindfulness OR psychoeducat* OR "problem solving" OR "digital intervention*" OR "internet-based intervention*" OR "web-based intervention*" OR "mobile intervention*" OR "mobile app*" OR "smartphone app*")<br>S8 AB (intervention* OR treatment* OR therap* OR psychotherap* OR "cognitive behavioral therap*" OR "cognitive behavioural therap*" OR mindfulness OR psychoeducat* OR "problem solving" OR "digital intervention*" OR "internet-based intervention*" OR "web-based intervention*" OR "mobile intervention*" OR "mobile app*" OR "smartphone app*")<br>S9 S7 OR S8<br>S10 TI (random* OR trial OR placebo OR waitlist OR "wait-list")<br>S11 AB (random* OR trial OR placebo OR waitlist OR "wait-list")<br>S12 S10 OR S11<br>S13 S3 AND S6 AND S9 AND S12
Search details: Last search date: 30 July 2026<br>Original yield: n = 53<br>Language: no filter<br>Publication date: no restriction<br>Publication type: no database filter; eligibility assessed during screening

**ProQuest Central**<br>**(via ProQuest) complete search strategy**
NOFT((<br>("university student*" OR "college student*" OR undergraduate* OR "graduate student*" OR postgraduate* OR "higher education student*" OR "tertiary education student*")<br>AND (depress* OR anxi* OR insomnia* OR "sleep quality" OR "sleep disturbance*" OR "sleep problem*" OR "sleep disorder*" OR "psychological distress")<br>AND (intervention* OR treatment* OR therap* OR psychotherap* OR "cognitive behavioral therap*" OR "cognitive behavioural therap*" OR mindfulness OR psychoeducat* OR "problem solving" OR "digital intervention*" OR "internet-based intervention*" OR "web-based intervention*" OR "mobile intervention*" OR "mobile app*" OR "smartphone app*")<br>AND (random* OR trial OR placebo OR waitlist OR "wait-list")<br>))
Search details: Last search date: 30 July 2026<br>Original yield: n = 402<br>Language: no filter<br>Publication date: no restriction<br>Publication type: no database filter; eligibility assessed during screening

Supplementary Table S3. Domain-level Cochrane Risk of Bias 2 (RoB 2) judgments for included studies.

Study	D1: Randomization	D2: Deviations	D3: Missing data	D4: Measurement	D5: Selection	Overall
[28] Bedford et al. (2018)	Low	Some concerns	High	Some concerns	Low	High
[29] Benjet et al. (2023)	Low	Some concerns	Some concerns	Low	Low	Some concerns
[30] Bruehlman-Senecal et al. (2020)	Low	Some concerns	Low	Some concerns	Low	Some concerns
[31] Hershner and O'Brien (2018)	Low	Low	Some concerns	Some concerns	Low	Some concerns
[32] Küchler et al. (2022)	Low	Low	Low	Some concerns	Low	Some concerns
[33] Lambert et al. (2025)	Low	Some concerns	High	Some concerns	Low	High
[34] Liu et al. (2024)	Low	Low	Low	Low	Low	Low
[35] Ponzo et al. (2020)	Low	High	High	High	Low	High
[36] Six et al. (2025)	Low	Low	Low	Some concerns	Low	Some concerns

Risk of bias was assessed using the Cochrane Risk of Bias 2 (RoB 2) tool across five domains. D1: bias arising from the randomization process; D2: bias due to deviations from intended interventions; D3: bias due to missing outcome data; D4: bias in measurement of the outcome; and D5: bias in selection of the reported result. Each domain was classified as low risk, some concerns, or high risk. Overall risk-of-bias judgments were determined according to the RoB 2 algorithm: a high-risk judgment in any domain resulted in an overall judgment of high risk, whereas one or more domains with some concerns, in the absence of any high-risk domain, resulted in an overall judgment of some concerns. Studies with low-risk judgments across all domains were classified as having an overall low risk of bias. Study numbers correspond to the reference list of the main manuscript.

Supplementary Table S4. Supporting rationales for domain-level Cochrane Risk of Bias 2 (RoB 2) judgments.

Study and source	Domain-specific supporting rationale
[28] Bedford et al. (2018)<br>doi:10.1016/j.beth.2017.11.010<br>Overall: High	D1 Randomization process - Low: Twenty-four student veterans were randomly allocated equally to ePST or minimal-contact control; no important baseline imbalance was reported.<br>D2 Deviations from intended interventions - Some concerns: Participants knew whether they received the six-session computerized treatment, and adherence and the handling of departures from assignment were not sufficiently detailed for the assignment-effect estimand.<br>D3 Missing outcome data - High: Incomplete repeated and posttreatment outcome data were substantial relative to this very small pilot sample, and the report did not provide sufficient evidence that missingness could not bias the estimated effect.<br>D4 Measurement of the outcome - Some concerns: Depression, PTSD, and insomnia were participant-reported in an unblinded comparison; knowledge of assignment could plausibly influence responses.<br>D5 Selection of the reported result - Low: The reported outcomes and assessment schedule corresponded to those described for the trial, with no evidence that the analyzed result was selected from multiple eligible estimates.<br>*High risk was driven by D3 (missing outcome data).*
[29] Benjet et al. (2023)<br>doi:10.1037/ccp0000846<br>Overall: Some concerns	D1 Randomization process - Low: A large, individually randomized three-arm trial was reported, with randomized allocation and no important imbalance that suggested a problem with the randomization process.<br>D2 Deviations from intended interventions - Some concerns: Participants and those delivering support could not be blinded, and engagement with guided and self-guided iCBT varied; these features leave some uncertainty about departures from the assigned intervention despite assignment-based analyses.<br>D3 Missing outcome data - Some concerns: Three-month outcome data were not available for every randomized participant. The analytic handling reduced concern, but the available information did not fully exclude bias from missing outcomes.<br>D4 Measurement of the outcome - Low: The same validated PHQ-9, GAD-7, and PHQ-ADS procedures were applied across groups, with no indication of differential outcome ascertainment.<br>D5 Selection of the reported result - Low: The trial was registered (NCT04780542), and the reported outcomes and analyses were consistent with the prespecified trial framework.<br>*Overall, some concerns arose from D2 and D3.*
[30] Bruehlman-Senecal et al. (2020)<br>doi:10.2196/21496<br>Overall: Some concerns	D1 Randomization process - Low: Qualtrics implemented stratified random allocation, and all randomized participants were included in the reported group structure without evidence of a randomization problem.<br>D2 Deviations from intended interventions - Some concerns: Participants and researchers were aware of app versus waitlist assignment, and engagement with the self-directed app could vary; this created some uncertainty about departures from the assigned intervention.<br>D3 Missing outcome data - Low: Week-4 data were available for 214 of 221 randomized participants (96.8%), so the small amount of missing data was unlikely to materially bias the estimate.<br>D4 Measurement of the outcome - Some concerns: PHQ-9, GAD-7, and sleep outcomes were self-reported by participants who knew their allocation, making an influence of treatment awareness on responses plausible.<br>D5 Selection of the reported result - Low: The trial was registered (NCT04164654), and the reported outcomes were consistent with the stated protocol and assessment schedule.<br>*Overall, some concerns arose from D2 and D4.*
[31] Hershner and O'Brien (2018)<br>doi:10.5664/jcsm.6974<br>Overall: Some concerns	D1 Randomization process - Low: College students were randomized to the online sleep-education intervention or control, and the report did not indicate an allocation problem or important baseline imbalance.<br>D2 Deviations from intended interventions - Low: The assigned intervention was a brief, standardized online program; no crossover or differential cointervention that would materially alter the assignment contrast was reported.<br>D3 Missing outcome data - Some concerns: Eight-week analyses relied on respondents to repeated surveys, and the available report did not provide a sufficiently robust sensitivity analysis to rule out bias from incomplete follow-up.<br>D4 Measurement of the outcome - Some concerns: Sleep quality, sleepiness, sleep hygiene, and depression were self-reported in a comparison in which participants knew whether they had received the online intervention.<br>D5 Selection of the reported result - Low: The stated outcomes and time points were reported consistently, with no evidence of selective choice among multiple eligible analyses for the assessed result.<br>*Overall, some concerns arose from D3 and D4.*

Study and source	Domain-specific supporting rationale
[32] Küchler et al. (2022)<br>doi:10.1007/s12671-022-01949-w<br>Overall: Some concerns	D1 Randomization process - Low: An independent researcher used block randomization with sealed allocation; 149 of 150 randomized participants entered the intention-to-treat analysis.<br>D2 Deviations from intended interventions - Low: The intervention was evaluated according to assignment, and no material crossover or unbalanced cointervention was reported.<br>D3 Missing outcome data - Low: Although attrition was higher in the intervention group (16% versus 4%), the intention-to-treat analysis used multiple imputation across 20 data sets, reducing the likelihood of material bias.<br>D4 Measurement of the outcome - Some concerns: The trial was open label and PHQ-9 and GAD-7 outcomes were participant-reported, so awareness of intervention assignment could influence responses.<br>D5 Selection of the reported result - Low: The registered trial and published protocol specified the principal outcomes and analyses, and the report was consistent with them.<br>*Overall, some concerns arose from D4.*
[33] Lambert et al. (2025)<br>doi:10.2196/58164<br>Overall: High	D1 Randomization process - Low: Qualtrics performed automated simple randomization, the research team was blind to allocation, and the randomized groups were broadly comparable.<br>D2 Deviations from intended interventions - Some concerns: Participants knew their assignment, and a Qualtrics delivery error disrupted scheduled follow-up for a subset of participants; these departures created some uncertainty for the assignment-effect estimate.<br>D3 Missing outcome data - High: Only 147 of 468 randomized participants completed the 2-week follow-up and 118 completed the 4-week follow-up. The primary complete-case result was sensitive to multiple imputation: the 2-week depression difference changed from $P = .03$ to $P = .07$.<br>D4 Measurement of the outcome - Some concerns: PHQ-9 and GAD-7 were self-reported by participants who knew whether they had completed COMET or the attention control, so responses could be influenced by assignment awareness.<br>D5 Selection of the reported result - Low: The article transparently reported the late registration, protocol changes, complete-case analysis, and imputation sensitivity analysis; no selective choice among eligible depression results was identified.<br>*High risk was driven by D3 (missing outcome data).*
[34] Liu et al. (2024)<br>doi:10.1016/j.psychres.2023.115671<br>Overall: Low	D1 Randomization process - Low: The report described randomized allocation of undergraduates with major depressive disorder to MBCT or the control condition, without evidence of a compromised sequence or important baseline imbalance.<br>D2 Deviations from intended interventions - Low: The structured eight-week intervention was evaluated according to assigned group, and no material crossover or differential cointervention was reported.<br>D3 Missing outcome data - Low: Outcome completeness and the reported handling of participant data did not indicate missingness sufficient to materially distort the effect estimate.<br>D4 Measurement of the outcome - Low: The same validated PHQ-9, GAD-7, and PSQI procedures and assessment times were used across groups, with no indication of differential outcome ascertainment.<br>D5 Selection of the reported result - Low: The reported outcomes and analyses corresponded to the stated trial objectives, and no evidence of selective result reporting was identified.<br>*All five domains were judged low risk.*
[35] Ponzo et al. (2020)<br>doi:10.2196/17767<br>Overall: High	D1 Randomization process - Low: Allocation used minimization based on age, sex, anxiety, and stress, with a random first allocation and an R-generated random-number list; no important baseline imbalance was reported.<br>D2 Deviations from intended interventions - High: The trial was unblinded and excluded randomized participants who returned to normal-range symptoms at baseline, did not download or open the app, or did not complete all questionnaires. The analyzed contrast therefore did not preserve assignment irrespective of adherence.<br>D3 Missing outcome data - High: At the final assessment, only 59 of 130 intervention participants and 64 of 132 controls completed the questionnaire. Complete-questionnaire exclusions and substantial attrition could plausibly bias the estimate.<br>D4 Measurement of the outcome - High: PHQ-9 and other mental health outcomes were self-reported in an unblinded app-versus-waitlist comparison, and knowledge of assignment could plausibly influence outcome reports.<br>D5 Selection of the reported result - Low: The study was preregistered on OSF (2zd45), and the stated outcome measures and assessment schedule were reported without evidence of selective result choice.<br>*High risk was driven jointly by D2, D3, and D4.*

Study and source	Domain-specific supporting rationale
[36] Six et al. (2025)<br>doi:10.2196/67381<br>Overall: Some concerns	D1 Randomization process - Low: The analyzed cohort of 209 students was randomized across four virtual-agent feature conditions, with no evidence that assignment was predictable or produced an important baseline imbalance.<br>D2 Deviations from intended interventions - Low: The assigned conversational and animation features were delivered through the same app framework, and no crossover or differential cointervention affecting the randomized feature contrasts was reported.<br>D3 Missing outcome data - Low: Baseline and two-week PHQ-8 data were available for the analyzed randomized cohort; the report did not indicate material differential missingness within that cohort.<br>D4 Measurement of the outcome - Some concerns: Participants experienced the randomized app features and self-reported PHQ-8 outcomes, so they were aware of salient intervention characteristics that could influence responses.<br>D5 Selection of the reported result - Low: The quantitative hypotheses, outcomes, and factorial analyses were stated and reported consistently, with no evidence that the assessed result was selected from multiple eligible estimates.<br>*Overall, some concerns arose from D4.*

Supplementary Table S5. Outcome completion and attrition at the study level.

Study	Randomized/analysis denominator	Outcome completion	Attrition and reasons
[28] Bedford et al. (2018)	N=24; ePST 12, minimal-contact control 12.	Week-6/posttreatment denominator NR in the accessible report.	Number and reasons for losses NR.
[29] Benjet et al. (2023)	N=1,319; guided i-CBT 445, self-guided i-CBT 439, TAU 435.	3 months: 291/445 (65.4%), 273/439 (62.2%), and 335/435 (77.0%), respectively; total 899/1,319 (68.2%).	Lost: 154, 166, and 100, respectively (420 total). Individual reasons NR; repeated email, messaging, and telephone contact was used to reduce nonresponse.
[30] Bruehlman-Senecal et al. (2020)	N=221; Nod 100, delayed access 121.	Week 4: 97/100 (97.0%) and 117/121 (96.7%); total 214/221 (96.8%).	Lost: 3 and 4, respectively; reasons NR.
[31] Hershner and O'Brien (2018)	Invitation-level allocation: 1,200/group. Baseline respondents: intervention 254; control 295 in text (297 in flow diagram).	Week 8: intervention 161/254 (63.4%); control 197/295 (66.8%; 197/297 [66.3%] using flow-diagram denominator).	Losses from text denominators: 93 and 98. Reasons NR; the control baseline denominator is internally inconsistent in the report.
[32] Küchler et al. (2022)	N=150 enrolled; 1 withdrew consent; ITT N=149 (intervention 74, waitlist 75).	Week 6: 62/74 (83.8%) and 72/75 (96.0%); total 134/149 (89.9%).	Lost after ITT entry: 12 and 3, respectively; individual reasons NR.
[33] Lambert et al. (2025)	N=468; COMET 239, attention control 229.	Postintervention: 203/239 (84.9%) and 204/229 (89.1%). Overall follow-up: 147/468 (31.4%) at 2 weeks; 118/468 (25.2%) at 4 weeks.	Lost: 321 at 2 weeks and 350 at 4 weeks. A Qualtrics error delayed/missed automated invitations for part of the cohort; other individual reasons NR.
[34] Liu et al. (2024)	N=60; MBCT 30, waitlist 30.	Week 8: 26/30 (86.7%) and 30/30 (100%); total 56/60 (93.3%).	Four MBCT losses: 3 declined further participation and 1 studied abroad.
[35] Ponzo et al. (2020)	N=262; BioBase 130, waitlist 132.	Week 6/final: 59/130 (45.4%) and 64/132 (48.5%); total 123/262 (46.9%).	Lost/not analyzed: 71 and 68. Thirty-one returned to normal-range symptoms between screening and baseline (15/16); remaining exclusions included non-initiation and incomplete questionnaires, without counts by reason.
[36] Six et al. (2025)	The report describes N=209 as both randomized and study completers across four active app conditions; arm-level n NR.	Two-week analysis: N=209; retention relative to the initially randomized cohort cannot be calculated.	Data were excluded for <2 CBT modules, >1 failed attention check, or no postintervention survey; initial N and counts by reason NR.

Outcome completion refers to the principal postintervention/follow-up point reported by each trial; it is not equivalent to intervention adherence. Percentages were calculated from the denominators shown when the source reported counts.

Supplementary Table S6. Intervention adherence and engagement at the study level.

Study	Definition/measurement	Quantitative result
[28] Bedford et al. (2018)	Six weekly ePST sessions were planned; no adherence threshold was stated.	Sessions attended/completed and denominator NR.
[29] Benjet et al. (2023)	Per protocol: ≥3 hours online (approximately 25 minutes × 7 core modules).	Guided: 124/445 (27.9%) reached ≥3 hours; 272/445 (61.1%) used <3 hours; 49/445 (11.0%) did not enroll. Self-guided: 47/439 (10.7%), 332/439 (75.6%), and 60/439 (13.7%), respectively.
[30] Bruehlman-Senecal et al. (2020)	Self-directed use; no minimum dose. Engagement was measured by account creation, pages accessed, challenges completed, and reflections viewed.	96/100 (96.0%) created an account by week 4. Among these 96: mean 36.69 pages (SD 38.50; median 23, IQR 12–43), 0.89 challenges, and 1.13 reflections.
[31] Hershner and O'Brien (2018)	Single approximately 20-minute website; no prespecified adherence threshold. Use was self-reported at week 8.	122/160 respondents (76.3%) reported using the website; 38/160 (23.8%) reported no use; 1/161 had missing use data.
[32] Küchler et al. (2022)	Adherent/per protocol: completion of ≥4 of 5 modules by week 6 (≥80%).	48/74 (64.9%) completed ≥4 modules; mean 3.57/5 (SD 1.55). Module 1: 70/74 (94.6%); all 5 modules: 27/74 (36.5%).
[33] Lambert et al. (2025)	Completion of all four COMET components in the single intervention session.	212/239 (88.7%) completed COMET. In the control arm, 213/229 (93.0%) completed the attention-control task.
[34] Liu et al. (2024)	Completer: attendance at ≥4 of 8 MBCT sessions; partial attendance: <4 sessions.	Among 26 analyzed MBCT participants, median attendance was 8 sessions (IQR 7–8). Exact numbers above/below the ≥4-session threshold NR.
[35] Ponzo et al. (2020)	Daily app use for ≥5 minutes was encouraged; no dichotomous adherence threshold was specified.	Among 59 final BioBase participants: mean 21.9/29 use days (median 26; IQR 13; range 2–29) and mean 5.33 minutes/day (SD 5.03; range 2.13–28.68).
[36] Six et al. (2025)	Use every other day for 2 weeks; ≥8 uses defined full completion. Analytic data required ≥2 CBT modules.	Number reaching ≥8 uses NR. The N=209 analytic cohort had completed ≥2 modules by definition; app-use counts were not reported.

Because constructs, thresholds, denominators, and collection methods differed; adherence estimates were not pooled.

Supplementary Table S7. Adverse-event and deterioration reporting at the study level.

Study	Ascertainment/reporting method	Reported events and interpretation
[28] Bedford et al. (2018)	Adverse-event ascertainment NR.	Event counts NR; safety not assessable.
[29] Benjet et al. (2023)	Recent suicidal ideation with intent was an exclusion criterion; positive baseline screens were immediately referred to a university clinical liaison. No post-randomization adverse-event surveillance was described.	Intervention-emergent event counts NR; safety not assessable.
[30] Bruehlman-Senecal et al. (2020)	Adverse-event ascertainment NR.	Event counts NR; safety not assessable.
[31] Hershner and O'Brien (2018)	Adverse-event ascertainment NR.	Event counts NR; safety not assessable.
[32] Küchler et al. (2022)	Reliable deterioration in mindfulness was defined as an FMI decrease >4.91 points. This was an outcome-based deterioration signal, not adverse-event surveillance; the authors stated that broader negative effects were not explored.	Reliable deterioration: intervention 1/74 (1.4%); waitlist 8/75 (10.7%). Other event counts NR; broader safety not assessable.
[33] Lambert et al. (2025)	A PHQ-9 item 9 score >0 triggered an automated pop-up with sources of help; participants were told responses were not routinely monitored. No systematic adverse-event surveillance was described.	Suicidal-ideation and other event counts NR; safety not assessable.
[34] Liu et al. (2024)	Adverse-event ascertainment NR.	Event counts NR; safety not assessable.
[35] Ponzo et al. (2020)	Adverse-event ascertainment NR.	Event counts NR; safety not assessable.
[36] Six et al. (2025)	Adverse-event ascertainment NR.	Event counts NR; safety not assessable.

AE: adverse event; CBT: cognitive behavioral therapy; FMI: Freiburg Mindfulness Inventory; i-CBT: internet-delivered cognitive behavioral therapy; IQR: interquartile range; ITT: intention to treat; MBCT: mindfulness-based cognitive therapy; NR: not reported; PHQ-9: Patient Health Questionnaire-9; SD: standard deviation; TAU: treatment as usual. NR indicates missing reporting, not absence of events.

Supplementary Table S8. Leave-one-out sensitivity analyses for depressive symptoms, anxiety, and sleep quality.

Outcome	Omitted study	k	Effect measure	Pooled effect	95% CI	p value	τ²	I² (%)
Depressive symptoms (PHQ-9)	Benjet et al.	6	MD	2.43	0.93 to 3.94	0.009	1.66	80.9
Depressive symptoms (PHQ-9)	Bruehlman-Senecal et al.	6	MD	2.34	0.76 to 3.92	0.013	1.90	83.4
Depressive symptoms (PHQ-9)	Hershner and O'Brien	6	MD	2.31	0.70 to 3.93	0.014	1.96	83.3
Depressive symptoms (PHQ-9)	Küchler et al.	6	MD	2.05	0.48 to 3.63	0.020	1.86	82.5
Depressive symptoms (PHQ-9)	Lambert et al.	6	MD	2.36	0.79 to 3.94	0.012	1.86	82.6
Depressive symptoms (PHQ-9)	Liu et al.	6	MD	1.75	0.88 to 2.62	0.004	0.29	41.6
Depressive symptoms (PHQ-9)	Ponzo et al.	6	MD	2.23	0.61 to 3.86	0.017	2.03	84.4
Anxiety (GAD-7)	Benjet et al.	4	MD	2.27	-1.19 to 5.72	0.128	4.40	93.5
Anxiety (GAD-7)	Bruehlman-Senecal et al.	4	MD	2.16	-1.39 to 5.71	0.148	4.68	94.1
Anxiety (GAD-7)	Küchler et al.	4	MD	2.02	-1.61 to 5.65	0.175	4.89	94.1
Anxiety (GAD-7)	Lambert et al.	4	MD	2.37	-0.94 to 5.68	0.107	3.99	92.2
Anxiety (GAD-7)	Liu et al.	4	MD	1.06	0.08 to 2.05	0.041	0.04	10.2
Sleep quality	Bruehlman-Senecal et al.	2	Hedges' g	1.86	-17.25 to 20.96	0.433	4.43	97.9
Sleep quality	Hershner and O'Brien	2	Hedges' g	1.79	-18.23 to 21.81	0.460	4.87	98.0
Sleep quality	Liu et al.	2	Hedges' g	0.33	-0.71 to 1.37	0.155	0.00	0.0

I^2: percentage of total variability attributable to between-study heterogeneity; τ^2: estimated between-study variance; CI: confidence interval; MD: mean difference in scale points. Positive MD values favor the intervention. Random-effects models were fitted using restricted maximum likelihood (REML) estimation with the modified Hartung–Knapp adjustment. p values correspond to tests of the pooled effect. Leave-one-out analyses for sleep quality that resulted in fewer than three remaining studies were considered exploratory.
